# Soloxolone *para*-methylanilide effectively suppresses aggressive phenotype of glioblastoma cells including TGF-β1-induced glial-mesenchymal transition *in vitro* and inhibits growth of U87 glioblastoma xenografts in mice

**DOI:** 10.3389/fphar.2024.1428924

**Published:** 2024-07-29

**Authors:** Kirill V. Odarenko, Aleksandra V. Sen’kova, Oksana V. Salomatina, Oleg V. Markov, Nariman F. Salakhutdinov, Marina A. Zenkova, Andrey V. Markov

**Affiliations:** ^1^ Institute of Chemical Biology and Fundamental Medicine, Siberian Branch of the Russian Academy of Sciences, Novosibirsk, Russia; ^2^ N.N. Vorozhtsov Novosibirsk Institute of Organic Chemistry, Siberian Branch of the Russian Academy of Sciences, Novosibirsk, Russia

**Keywords:** brain cancer, triterpenoid, mesenchymal transition, cancer stem cell, combination therapy

## Abstract

Soloxolone amides are semisynthetic triterpenoids that can cross the blood-brain barrier and inhibit glioblastoma growth both *in vitro* and *in vivo*. Here we investigate the impact of these compounds on processes associated with glioblastoma invasiveness and therapy resistance. Screening of soloxolone amides against glioblastoma cells revealed the ability of compound **7** (soloxolone *para*-methylanilide) to inhibit transforming growth factor-beta 1 (TGF-β1)-induced glial-mesenchymal transition Compound **7** inhibited morphological changes, wound healing, transwell migration, and expression of mesenchymal markers (N-cadherin, fibronectin, Slug) in TGF-β1-induced U87 and U118 glioblastoma cells, while restoring their adhesiveness. Confocal microscopy and molecular docking showed that **7** reduced SMAD2/3 nuclear translocation probably by direct interaction with the TGF-β type I and type II receptors (TβRI/II). In addition, **7** suppressed stemness of glioblastoma cells as evidenced by inhibition of colony forming ability, spheroid growth, and aldehyde dehydrogenase (ALDH) activity. Furthermore, **7** exhibited a synergistic effect with temozolomide (TMZ) on glioblastoma cell viability. Using N-acetyl-L-cysteine (NAC) and flow cytometry analysis of Annexin V-FITC-, propidium iodide-, and DCFDA-stained cells, **7** was found to synergize the cytotoxicity of TMZ by inducing ROS-dependent apoptosis. Further *in vivo* studies showed that **7**, alone or in combination with TMZ, effectively suppressed the growth of U87 xenograft tumors in mice. Thus, **7** demonstrated promising potential as a component of combination therapy for glioblastoma, reducing its invasiveness and increasing its sensitivity to chemotherapy.

## 1 Introduction

Glioblastoma is the most aggressive type of brain tumor, with a median survival of 15 months. Its treatment efficacy remains low despite the combination of complete surgical resection, radiotherapy, and temozolomide (TMZ) chemotherapy, as well as significant advances in glioma diagnosis, including the development of deep learning-based approaches (Rui et al., 2023; Redlich et al., 2024). Complications of glioblastoma treatment include tumor infiltration into the brain tissue, high recurrence rates, and resistance to irradiation and TMZ (Rodríguez-Camacho et al., 2022). Therefore, new drugs are under developing to increase the susceptibility of glioblastoma to conventional therapy (Ali et al., 2020; Tomar et al., 2021).

Glioblastoma exhibits intratumoral heterogeneity, which is an important consideration for drug development. The transcriptomic subtypes of glioblastoma, namely, classical, proneural, and mesenchymal, can coexist within a single tumor (Becker et al., 2021). As the disease progresses, there is a shift toward a mesenchymal phenotype, resulting in increased tumor invasive potential. This process is similar to the epithelial-mesenchymal transition (EMT) in carcinomas, but it differs in regulation and manifestation, and is therefore termed glial-mesenchymal transition (GMT) (Lai et al., 2024). Glioblastoma recurrence is associated with a subset of glioblastoma stem cells (GSCs) that divide asymmetrically, thereby restoring the tumor cell population suppressed by therapy (Xie et al., 2022). In addition, both GMT and GSCs have been found to enhance glioblastoma resistance to radiotherapy and TMZ treatment (Wang et al., 2021; Lai et al., 2024).

Recent reviews suggest that several compounds with anti-GMT and anti-GSC properties have been developed as adjuncts to glioblastoma therapy (Hersh et al., 2022; Lai et al., 2024). Although these compounds have exhibited promising antitumor activity *in vitro* and *in vivo*, their impact on patient survival in clinical trials has been limited (Hersh et al., 2022; Lai et al., 2024). Therefore, further researches are required to fully realize the potential of such therapeutic agents. One of the main challenges in this field is crossing the blood-brain barrier, which limits drug delivery due to tight junctions and multidrug efflux transporters (Sprowls et al., 2019). Possible solutions include disrupting the blood-brain barrier or developing compounds capable of both crossing into brain parenchyma and exhibiting anti-GMT and anti-GSC activities.

**FIGURE 1:**
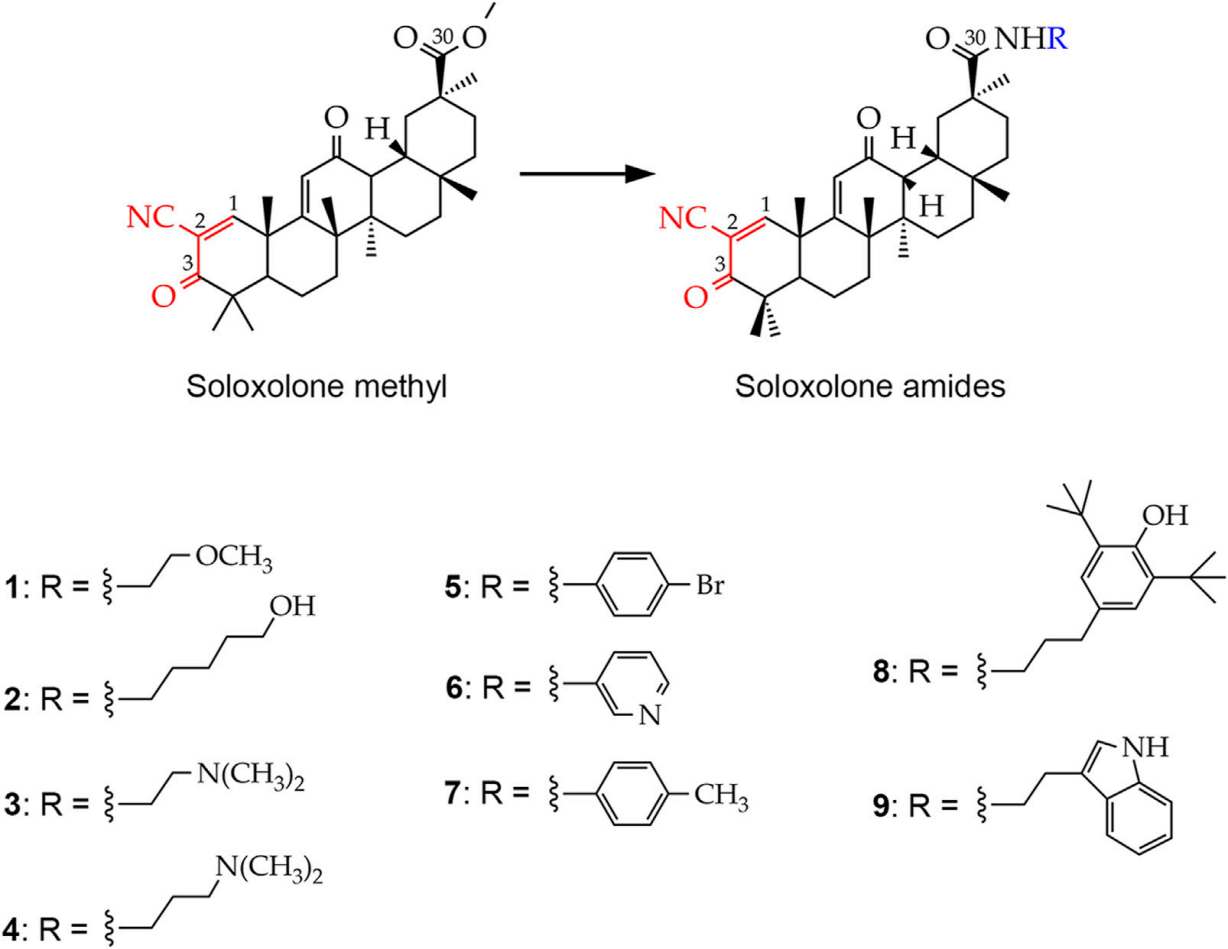
Chemical structures of the soloxolone amides: N-(20-hydroxyethyl)-2-cyano-3,12-dioxo-18βH-olean-9(11),1(2)-dien-30-amide (**1**), N-(5′-hydroxypentyl)-2-cyano-3,12-dioxo-18βH-olean-9(11),1(2)-dien-30-amide (**2**), N-(2′-(dimethylamino)ethyl)-2-cyano-3,12-dioxo-18βH-olean-9(11),1(2)-dien-30-amide (**3**), N-(3′-(dimethylamino)propyl)-2-cyano-3,12-dioxo-18βH-olean-9(11),1(2)-dien-30-amide (**4**), N-(4′-bromophenyl)-2-cyano-3,12-dioxo-18βH-olean-9(11),1(2)-dien-30-amide (**5**), N-(pyridin-3-yl)-2-cyano-3,12-dioxo-18βH-olean-9(11),1(2)-dien-30-amide (**6**), N-*p*-tolyl-2-cyano-3,12-dioxo-18βH-olean-9(11),1(2)-dien-30-amide (**7**), N-(3′-(3″,5″-di-*tert*-butyl-4″-hydroxyphenyl)propyl)-2-cyano-3,12-dioxo-18βH-olean-9(11),1(2)-dien-30- amide (**8**), N-(2′-(1H-indol-2-yl)-ethyl)-2-cyano-3,12-dioxo-18βH-olean-9(11),1(2)-dien-30-amide (**9**). The cyanoenone pharmacophore group, inherited by soloxolone amides from their predecessor soloxolone methyl (SM), is highlighted in red. The blue color indicates the position where the depicted amide moieties bind to the soloxolone scaffold.

Our research team has previously developed soloxolone methyl (SM), a semisynthetic triterpenoid bearing a cyanoenone pharmacophore group that determines its high antitumor activity (Logashenko et al., 2011) (Figure 1). It was shown that SM induced cervical carcinoma cell death via endoplasmic reticulum stress, probably due to its direct interaction with SERCA2 and GRP94 (Markov et al., 2019; Alper et al., 2021). In addition, SM effectively inhibited the metastatic potency of lung adenocarcinoma and melanoma cells by suppressing EMT, hypothetically through its interactions with MMP-2/9 and JNK1, as determined by network pharmacology analysis (Markov et al., 2020).

To improve the pharmacological properties of SM, including poor blood-brain barrier permeability, a series of its derivatives containing different amide moieties at the C-30 position have been developed (Figure 1). Soloxolone amides were found not only to cross the blood-brain barrier, but also to inhibit glioblastoma growth by inducing ROS-dependent apoptosis (Markov et al., 2022b). Our recent studies show that soloxolone tryptamide (compound **9**) inhibits the motility and spheroid growth of glioblastoma cells, reducing their invasive potential and stemness, respectively (Markov et al., 2022b; Markov et al., 2023). In addition, it acts synergistically with TMZ to reduce glioblastoma cell viability (Markov et al., 2023). Based on these findings, it is worth exploring the soloxolone amide library to identify potential GMT and GSC inhibitors that could be used as a compound of combination regimens to improve outcomes of conventional therapy.

Here we report the GMT-modulating activity of soloxolone amides in human and murine glioblastoma cells. The effects of hit compound **7** on cell migration, adhesion, GMT marker expression, stemness, and TMZ cytotoxicity were investigated using three glioblastoma cell lines. Reactive oxygen species (ROS), TGF-β1 signaling, and aldehyde dehydrogenase (ALDH) activity were examined to gain mechanistic insight into the activity of compound **7**. In addition, the efficacy of **7** as an anti-glioblastoma agent was verified in both mono- and TMZ-combined regimens in a mouse xenograft model. Our results support the rationale for further investigation of soloxolone amides as potential drug candidates that affect glioblastoma invasion and stemness.

## 2 Materials and methods

### 2.1 Reagents and antibodies

Soloxolone amides were synthesized as previously described (Markov et al., 2022b) and were dissolved in DMSO at a concentration of 10 mM and stored at −20 °C before use. Human recombinant TGF-β1 (CYT-716) was purchased from ProSpec-Tany TechnoGene Ltd. (Ness-Ziona, Israel). The rabbit anti-Smad2/Smad3 antibody (ab202445) and the goat anti-rabbit IgG antibody conjugated with Alexa Fluor 488 (ab150077) were obtained from Abcam (Cambridge, MA, USA). Diamidino-2-phenylindole (DAPI) was purchased from Thermo Fisher Scientific (Rockford, IL, United States).

### 2.2 Cell cultures

Human glioblastoma U87 and U118 and mouse glioblastoma EPNT-5 cell lines were obtained from the Russian Culture Collection (Institute of Cytology of the Russian Academy of Sciences (RAS), St. Petersburg, Russia). Human non-transformed hFF3 foreskin fibroblasts were kindly provided by Dr. Olga A. Koval (Institute of Chemical Biology and Fundamental Medicine of the Siberian Branch of RAS, Novosibirsk, Russia). All cells were maintained in DMEM (U87, U118, EPNT-5) or IMDM (hFF3) medium supplemented with 10% fetal bovine serum (FBS; Dia-M, Moscow, Russia) and 1% antibiotic-antimycotic solution (100 U/mL penicillin, 100 μg/mL streptomycin, 0.25 μg/mL amphotericin B; Central Drug House Pvt. Ltd., New Delhi, India) at 37 °C and 5% CO_2_ in a humidified atmosphere.

### 2.3 Mice

Six-to-eight-week-old female athymic nude mice with average weight of 22–24 g were purchased from the Center for Genetic Resources of Laboratory Animals at the Institute of Cytology and Genetics of the Siberian Branch of the Russian Academy of Sciences (SB RAS) (Novosibirsk, Russia). Animals were kept in plastic cages (5-6 animals per cage) under a normal daylight schedule in temperature-controlled, specific pathogen-free conditions. Water and food were provided *ad libitum*. All animal procedures were carried out in strict accordance with the European Communities Council Directive 86/609/CEE. The experimental protocol was approved by the Committee on the Ethics of Animal Experiments at the Institute of Cytology and Genetics SB RAS (protocol no. 56 of 10 August 2019). Subcutaneous and intraperitoneal injections were performed using a needle with a diameter of no more than 0.25 mm. The stressful effect lasted no longer than the time interval required for subcutaneous or intraperitoneal injection. Mice were euthanized at the end of the experiment under isoflurane anesthesia using a gas mixture containing 3% isoflurane +97% air at a flow rate of 2 L/min.

### 2.4 Cell viability assay

Cells were seeded in quadruplicate in 96-well plates at 10,000 cells/well and allowed to adhere overnight. The medium was then replaced with serum-free DMEM containing increasing concentrations of soloxolone amides for 48 h. After incubation, 3-(4,5-dimethylthiazol-2-yl)-2,5-diphenyltetrazolium bromide (MTT dye) was added at a final concentration of 0.5 mg/mL and left for 2 h. The resulting formazan crystals were dissolved in DMSO and the optical density was measured at 570 nm using a Multiscan RC plate reader (Thermo LabSystems, Helsinki, Finland).

For synergy analysis, cell viability was assessed after 72-h incubation with each dose combination of compound **7** (0–400 nM) and TMZ (0–500 μM) using the MTT assay. The drug synergy score was calculated for the dose-response matrix according to the highest single-agent (HSA) model using the SynergyFinder + web application (https://www.synergyfinder.org/).

### 2.5 Cell adhesion assay

Adhesiveness of glioblastoma cells was assessed by two independent methods. First, cell adhesion to culture plastic was tested using a trypsin treatment assay, as previously described (Sava et al., 2004; Mazuryk et al., 2015; van Dijk et al., 2017). Briefly, U87 and U118 cells (10,000 cells per well of a 96-well plate, n = 4) were cultured with TGF-β1 (50 ng/mL) and non-toxic concentrations of soloxolone amides for 48 h and then treated with a 1:20 dilution of TrypLE Express (Gibco, USA) in PBS for 3 min at 37°C. Non-adherent cells were then removed by a triple PBS wash, and the remaining adherent cells were fixed with 4% formaldehyde and stained with crystal violet dye (0.1% w/v). Images were captured using the iBright 1,500 Imaging System (Invitrogen, USA), and the cell-occupied area was calculated using ImageJ. Alternatively, the number of adherent cells was quantified using the MTT assay.

Second, to assess cell adhesion to the extracellular matrix (ECM), an ECM adhesion assay was performed according to previously published method (Guan et al., 2015; Hu et al., 2019). Briefly, U87 cells were pretreated with TGF-β1 (50 ng/mL) and compound **7** (50 nM) for 48 h, and then seeded in 96-well plates coated with Matrigel (BD Biosciences, Bedford, MA, USA) at a concentration of 30,000 cells for 1 h (n = 3). After that, the wells were washed to remove non-adherent cells, adherent cells were fixed with formaldehyde, stained with crystal violet dye, and analyzed as described above.

### 2.6 Assessment of cell morphology

After seeding at a concentration of 5,000 cells/well in triplicate in 96-well plates and incubating overnight, U87 and U118 cells were exposed to TGF-β1 (50 ng/mL) and non-toxic concentrations of soloxolone amides for 48 h. An EVOS XL Core microscope with an integrated CMOS camera (Thermo Fisher Scientific, USA) was then used to examine cell morphology at ×20 magnification. Aspect ratio (AR) and area were quantified for at least 150 cells in each experimental group using ImageJ software (NIH, USA).

### 2.7 Assessment of cell migration

To perform transwell migration, U87 cells were pretreated with TGF-β1 (50 ng/mL) and compound **7** (50 nM) for 48 h. Subsequently, 250,000 cells were placed in the upper chamber of the CIM-16 xCELLigence plate in serum-free DMEM supplemented with compound **7** and TGF-β1 (n = 4), while the lower chamber was filled with DMEM containing 10% FBS. Cell migration was monitored for 72 h using the xCELLigence RTCA DP system (ACEA Biosciences Inc., San Diego, CA, USA). The electrodes placed under the porous membrane detected changes in resistance, which were then converted into cell indices proportional to the number of migrating cells.

For the scratch assay, U118 cells were seeded in triplicate in 24-well plates at a concentration of 300,000 cells/well and allowed to adhere overnight. The cells were then scratched with a 10 μL plastic pipette tip, washed with PBS, and covered with serum-free DMEM containing TGF-β1 (50 ng/mL) and compound **7** (50 nM). Scratches were photographed at 0, 24, and 48 h using an EVOS XL Core microscope. Wound closure was determined by estimating the scratch area at each time point using ImageJ software and normalizing to the scratch area at 0 h.

### 2.8 Quantitative real-time polymerase chain reaction (qRT-PCR)

Total RNA was extracted using TRIzol Reagent (Ambion, Austin, TX, USA) according to the manufacturer’s protocol. Subsequently, the first strand cDNA was synthesized from 4 μg of total RNA using an oligo (dT)18 primers and M-MuLV-RH revertase (Biolabmix, Novosibirsk, Russia). Finally, RT-qPCR was performed using specific primers (Table 1) and BioMaster SYBR Blue reagent kit (Biolabmix, Novosibirsk, Russia). Relative expression was determined by normalization to the housekeeping gene *HPRT* using the 2^−ΔΔCT^ method (Livak and Schmittgen 2001).

**TABLE 1:** Primers used for qRT-PCR.

Gene	Forward primer	Reverse primer
*HPRT*	TAT​GGC​GAC​CCG​CAG​CCC​T	CAT​CTC​GAG​CAA​GAC​GTT​CAG
*CDH2 (N-cadherin)*	GCT​ACT​TTC​CTT​GCT​TCT​G	GAGGTAACACTTGAGGG
*FN1* (*Fibronectin*)	CCA​CTT​CCC​CTT​CCT​ATA​C	TCCCACTGATCTCCAATG
*SNAI2* (*Slug*)	GGAATATGTGAGCCTGG	TGACCTGTCTGCAAATG
*OLIG2*	AGC​TCC​TCA​AAT​CGC​ATC​C	AAA​AAG​GTC​ATC​GGG​CTC​TG

### 2.9 Colony formation assay

U87, U118 and EPNT-5 cells were seeded at a density of 200 cells/well in 96-well plates and treated with increasing concentrations of compound **7** (0–1,000 nM) dissolved in 10% FBS-containing DMEM (n = 5). On day 10, colonies were fixed with 4% formaldehyde and stained with crystal violet dye (0.1% w/v). Images were captured using the iBright 1,500 Imaging System (Invitrogen, USA) and the area of colonies with a minimum diameter of 100 µm was calculated using ImageJ. In the case of overlapping colonies, their total area was calculated. Finally, the area of the formed cell colonies was summed and normalized to the total colony area in the control.

### 2.10 Tumorsphere formation assay

For evaluation of spheroid growth, the bottom of the 96-well plate was covered with 1% agarose for non-adherent conditions. U87 cells (5,000 cells/well) were then seeded and allowed to form spheroids for 24 h, after which increasing concentrations of compound **7** (0–1,000 nM) were added (n = 8). Every other day of the 10-day experiment, fresh medium supplemented with compound **7** was added to half of the medium, and the spheroids were photographed using a camera-equipped ZEISS Primo Vert microscope (Carl Zeiss Microscopy GmbH, Jena, Germany). To assess the formation of secondary tumorspheres, cells were dissociated from primary tumorspheres after 4 days of compound **7** stimulation using TrypLE Express and were then seeded into agarose-coated 96-well plates (n = 8). On day 3, the resulting secondary spheroids were examined microscopically. Primary and secondary spheroid areas were quantified using ImageJ.

### 2.11 Flow cytometry

To assess ALDH activity, U87 cells were dissociated from spheroids using TrypLE Express after 4 days of induction with compound **7** (0–100 nM) (n = 3). Cells were then stained with the AldeRed ALDH Detection Assay (SCR150; EMD Millipore, Burlington, MA, USA) according to the manufacturer’s protocol. Experimental controls included U87 cells grown in 2D culture and cells treated with the ALDH inhibitor diethylaminobenzaldehyde (DEAB). Gates of ALDH-positive cells were set according to the inhibitory effect of DEAB on control cells.

To observe apoptosis and necrosis, U87 and U118 cells were stained with Annexin V-FITC and propidium iodide (ab14085; Abcam, Inc., Cambridge, MA, USA) after 48 h of incubation with compound **7** (0–2000 nM) according to the manufacturer’s protocol (n = 3). To quantify the total proportion of apoptotic cells in control and experimental groups, the events accumulated in the lower right quadrant (early apoptosis) and upper right quadrant (late apoptosis) were summarized.

Intracellular ROS levels were measured according to previously published studies (Han et al., 2008; Samandari-Bahraseman et al., 2023; Kopsida et al., 2024), using the DCFDA/H_2_DCFDA Cellular ROS Assay Kit (Abcam, Cambridge, United Kingdom). U87 cells were incubated with **7** and TMZ with or without NAC for 72 h (n = 3). Cells were then washed with PBS and incubated with 20 µM H_2_DCFDA for 30 min under standard conditions, followed by the removal of H_2_DCFDA-containing medium and double washing with PBS.

After staining, 10,000 cells per sample were analyzed on a NovoCyte flow cytometer using NovoExpress software (ACEA Biosciences, Inc., USA).

### 2.12 Immunofluorescence

U87 cells (150,000 cells/well) were allowed to attach to glass coverslips in 24-well plates prior to 48-h incubation with compound **7** (50 nM) under serum-free conditions, followed by stimulation with TGF-β1 (50 ng/mL) for 1 h. After fixation in 4% formaldehyde for 20 min, cells were incubated with primary antibodies against Smad2/3 (1:100, ab202445) for 1 h under permeabilizing conditions (0.1% Triton X100, 5 mg/mL BSA, 37°C) and Alexa Fluor 488-conjugated secondary antibodies (1:500, ab150077) for 1 h in the dark. Finally, nuclei were stained with DAPI (1 μg/mL) for 10 min and cells were mounted on glass slides with Fluoromount-G (Thermo Fisher Scientific, Rockford, IL, USA). Images were captured using a LSM710 confocal microscope (Zeiss, Oberkochen, Germany) at ×40 magnification. Nuclear translocation was quantified by measuring the ratio of nuclear to perinuclear intensity of Alexa Fluor 488 in CellProfiler software (Broad Institute, Cambridge, MA, USA) for 50 cells per experimental group (n = 3).

### 2.13 Molecular docking

The crystal structures of TGF-β receptors type I and II (TβRI and TβRII) were obtained from the Protein Data Bank (PDB IDs 5QIM and 5E91, respectively). Protein preparation included removal of co-crystallized ligands and solvent molecules, addition of polar hydrogen atoms, and calculation of Gasteiger charges in AutoDockTools v.1.5.7. The structure of compound **7** was created using MarvinSketch v.22.1, optimized with the MMFF94 force field in Avogadro v.1.2.0, and set free rotation using AutoDockTools. Finally, molecular docking was performed using AutoDock Vina v.1.2.5 with the parameters described in Table 2. The resulting docking models of compound **7** were analyzed using UCSF ChimeraX v.1.7.1. The inhibitors originally co-crystallized with TβRI and TβRII (PubChem CIDs 121411739 and 118988613, respectively) underwent the same processing as compound 7 and served as positive controls.

**TABLE 2:** Molecular docking parameters.

Protein target	PDB ID	Grid box					
		Coordinates			Size		
		x	y	z	x	y	z
TβRI	5QIM	4.272	9.573	5.503	22	16	16
TβRII	5E91	13.675	−0.527	3.375	18	16	20

### 2.14 Tumor transplantation and design of animal experiment

A xenograft model of tumor progression was induced by subcutaneous (s.c.) implantation of U87 glioblastoma cells (2×10^7^ cells/mL) suspended in 0.1 mL of saline buffer into the left flank of athymic nude mice. On day 4 after tumor transplantation, the mice were divided into four groups: (1) mice received intraperitoneal (i.p.) injections of 10% Tween-80 (vehicle) (n = 6); (2) mice received i.p. injections of compound **7** in 10% Tween-80 at a dose of 20 mg/kg (n = 5); (3) mice received i.p. injections of TMZ in saline buffer at a dose of 10 mg/kg (n = 5); (4) mice received combination therapy of compound **7** and TMZ at the same dosage and administration route (n = 6). Compound **7** was administered three times per week; in total seven injections were performed. TMZ was administered daily except weekends; in total eleven injections were performed. During the experiment, the tumor volumes were determined three times per week using caliper measurements and were calculated as V = (D × d^2^)/2, where D is the longest diameter of the tumor node and d is the shortest diameter of the tumor node perpendicular to D. Mice were sacrificed on day 19 after tumor transplantation and material (tumor nodes, livers, and kidneys) was collected for subsequent analysis.

### 2.15 Toxicity assessment

During the experiment, the general status and body weight of the animals were monitored. At the end of the experiment, livers and kidneys were collected and organ indices were calculated as (organ weight/body weight) × 100%, followed by the normalization of organ indices of experimental mice to organ indices of control mice.

### 2.16 Histology and immunohistochemistry

For the histological study, the tumor specimens were fixed in 10% neutral-buffered formalin (BioVitrum, Moscow, Russia), dehydrated in ascending ethanols and xylols and embedded in HISTOMIX paraffin (BioVitrum, Russia). The paraffin sections (3–4 µm) were sliced on a Microm HM 355S microtome (Thermo Fisher Scientific, Waltham, MA, USA) and stained with hematoxylin and eosin.

For the immunohistochemical study, the tumor sections were deparaffinized and rehydrated. Antigen retrieval was carried out after exposure in a microwave oven at 700 W. The samples were incubated with anti-Ki-67 (ab16667, Abcam, Boston, MA, USA) (n = 3), anti-GFAP (ab194325, Abcam, USA) (n = 3), and anti-N-cadherin (ab76011, Abcam, USA) (n = 3) primary antibodies according to the manufacturer’s protocol. Then, the sections were incubated with secondary horseradish peroxidase (HPR)-conjugated antibodies, exposed to the 3,30-diaminobenzidine (DAB) substrate (Rabbit Specific HRP/DAB (ABC) Detection IHC Kit, ab64261, Abcam, USA) and stained with Mayer’s hematoxylin.

Morphometric analysis of tumor sections included evaluation of the numerical density (Nv) of mitoses (n = 5–6) and Ki-67-positive cells (n = 3) indicating the number of particles studied in the square unit, 3.2 × 10^6^ μm^2^ in this case. Five to ten random fields were examined from the tumor specimens of three mice in each group, for a total of 15–30 test fields.

All the images were examined and scanned using an Axiostar Plus microscope equipped with an Axiocam MRc5 digital camera (ZEISS, Germany) at magnification of ×200 and ×400.

### 2.17 The association of *CDH2* and *GFAP* expression with pathogenesis of glioma/glioblastoma in patients

The analysis of the association of *CDH2* and *GFAP* expression levels with the survival rates of patients with recurrent glioma and mesenchymal-versus proneural-type of glioblastoma was performed based on the Chinese Glioma Genome Atlas (CCGA) and the Repository for Molecular BRAin Neoplasia DaTa (REMBRANDT), respectively, using GlioVis tool (Bowman et al., 2017).

### 2.18 Statistical analysis

Statistical analysis was performed using R Studio (2023.09.1 + 494) for R version 4.3.2 (*in vitro* experiments) and GraphPad Prism version 8.0.1 (GraphPad Software, San Diego, CA, USA) (*in vivo* experiment). Normality of the datasets was assessed using the Shapiro-Wilk criterion, and the appropriate statistical tests were performed using the “rstatix” package. Data from cell adhesion, cell morphology, transwell migration and immunofluorescence experiments were analyzed by Kruskal–Wallis test followed by Dunn’s post-hoc comparisons. Data from scratch assay, colony formation, tumorsphere formation and RT-qPCR experiments were analyzed by one-way ANOVA followed by Tukey’s post-hoc comparisons. Data from *in vivo* experiment were analyzed by unpaired Student’s t-test.

## 3 Results

### 3.1 Screening for inhibitory activity of soloxolone amides against glial-mesenchymal transition (GMT) and identification of a hit compound

**FIGURE 2:**
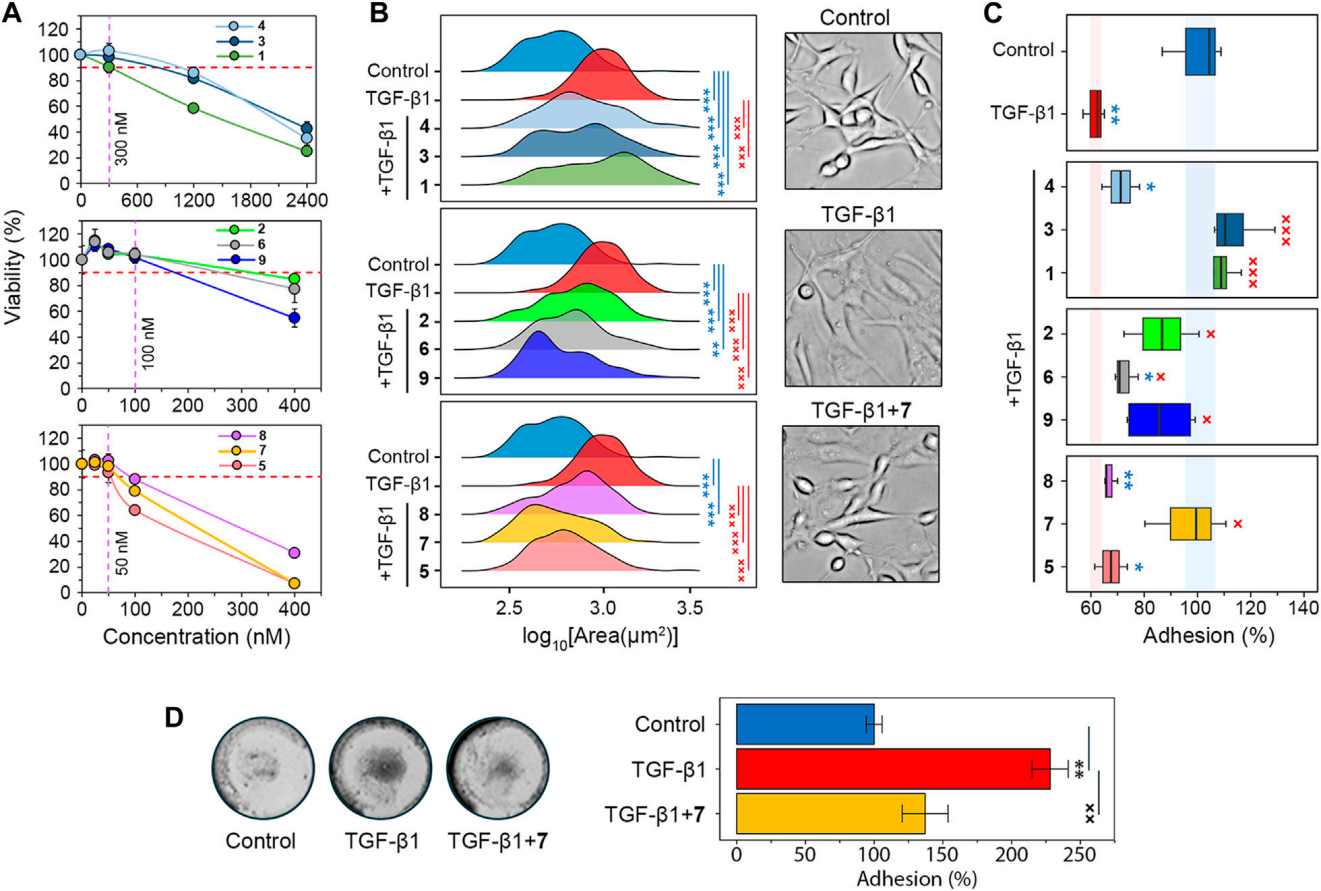
Screening of the soloxolone amide library for GMT inhibitory activity. **(A)** The cytotoxicity of soloxolone amides against U87 cells assessed by MTT assay at 48 h (n = 4). The purple dotted line shows non-toxic concentrations causing less than 10% cell death (red dotted line). **(B)** Size distribution of U87 cells after treatment with TGF-β1 (50 ng/mL) and soloxolone amides (non-toxic concentrations) for 48 h (n = 3). **(C)** Adhesion of U87 cells to culture plastic after treatment with TGF-β1 (50 ng/mL) and soloxolone amides (non-toxic concentrations) for 48 h (n = 4). **(D)** Adhesion of U87 cells to Matrigel after treatment with TGF-β1 (50 ng/mL) and compound **7** (50 nM) for 48 h (n = 3). Data in line graph are represented as mean ± standard deviation (SD). Statistical significance was calculated by comparison with the control (marked by *) or TGF-β1-treated group (marked by ×). */×, **/××, ***/××× indicate that *p*-values were less than 0.05, 0.01, and 0.001, respectively.

To identify the most promising soloxolone amide drug candidates, we screened their effect on glial-mesenchymal transition (GMT), a process associated with increased invasiveness of glioblastoma cells (Lai et al., 2024). First, the MTT assay was performed to determine low toxic concentrations of the compounds against U87 glioblastoma cells (Figure 2A). The soloxolone amides were classified into three groups based on their relative cytotoxicity after 48 h of incubation, in particular, group 1 (**1**, **3**, and **4**), group 2 (**2**, **6**, and **9**), and group 3 (**5**, **7**, and **8**) exhibited low, moderate, and high cytotoxicity, respectively. Based on the obtained cytotoxic profiles, non-toxic concentrations of compounds that reduced U87 cell viability by less than 10%, which were 300 nM, 100 nM, and 50 nM for compounds from group 1, 2, and 3, respectively, were selected for further screening experiments (Figure 2A). Interestingly, the most active soloxolone amides contain a benzene ring with lipophilic substituents, namely, methyl- (**5**), bromo- (**7**) and tert-butyl (**8**) groups, whereas compounds with moderate cytotoxicity bear moieties capable of forming H-bonds (**2**, **6**, **9**), being either H-donors due to the acidity of the O-H (**2**) and N-H (**9**) bonds or H-acceptor due to the basicity of the nitrogen atom of the pyridine ring (**6**). The least cytotoxic were compounds **1**, **3** and **4**, which have substituents that can also act as H-acceptors due to lone pairs of heteroatoms, but their ability to form H-bonds is much less pronounced compared to compound **6** of group 2.

Morphological changes, the weakening of cell-cell adhesion, and strengthening of cell-ECM adhesion are characteristic features of GMT in glioblastoma cells (Joseph et al., 2014; Iser et al., 2016; Jin et al., 2020; Zhitnyak et al., 2020). Microscopic examination showed that TGF-β1-induced U87 cells had elongated protrusions typical for mesenchymal cells (Joseph et al., 2014), which increased their size by 76% compared to control cells. All soloxolone amides shifted the distribution of cell size towards smaller values, and three compounds, **5, 7,** and **9**, restored their morphological characteristics to the control cells (Figure 2B).

To evaluate adhesion of U87 cells to culture plastic, cells were treated with TrypLE (a recombinant analog of trypsin) and the number of cells remaining attached to the culture plates after washing was quantified using crystal violet assay (Supplementary Figure S1A). Consistent with previous reports (Liu et al., 2015; Kang et al., 2019; Xu et al., 2022), incubation with the GMT-inducing cytokine TGF-β1 for 48 h reduced U87 adhesion by 38.5% (Figure 2C). The majority of tested soloxolone amides, except for **4**, **5** and **8**, markedly increased the resistance of TGF-β1-treated U87 cells to TrypLE-mediated cell dislodgement, with **1**, **3** and **7** restoring it to control levels (Figure 2C). To double-check the results obtained, the adhesion assay was repeated using the MTT assay to assess the amount of adherent cells by spectroscopy. The results obtained (Supplementary Figure S1B) were similar to those shown in Figure 2B, with **7** demonstrating one of the most pronounced pro-adhesive effects.

To further characterize the ability of **7** to modulate glioblastoma cell adhesion, its effect on U87 cell adhesion to Matrigel, which mimics the ECM, was examined. Surprisingly, in contrast to previous assays, TGF-β1 was found to significantly increase cell adhesion to Matrigel, whereas **7** effectively suppressed this induction to almost control levels (Figure 2D). We hypothesize that the opposite effect of compound **7** on the adhesion of U87 cells to culture plastic (Figure 2C) and Matrigel (Figure 2D) may be determined by differences in the key types of adhesion contacts in the adhesion assays used. In the case of the Matrigel adhesion assay, the main contribution to cell adhesion is made by cell-matrix contacts, whereas in the case of TrypLE-mediated cell detachment, a significant role in cell adhesion is played by cell-cell interactions, the strength of which decreases at GMT (Zhitnyak et al., 2020). In any case, our results clearly indicate the ability of compound **7** to block the effect of TGF-β1 on the adhesiveness of glioblastoma cells. Based on these findings, soloxolone *para*-methylanilide (compound **7**) was revealed as a hit compound that inhibited both morphological changes and tumor cell adhesiveness during GMT.

### 3.2 Compound 7 inhibited GMT in TGF-β1-induced glioblastoma cells

**FIGURE 3:**
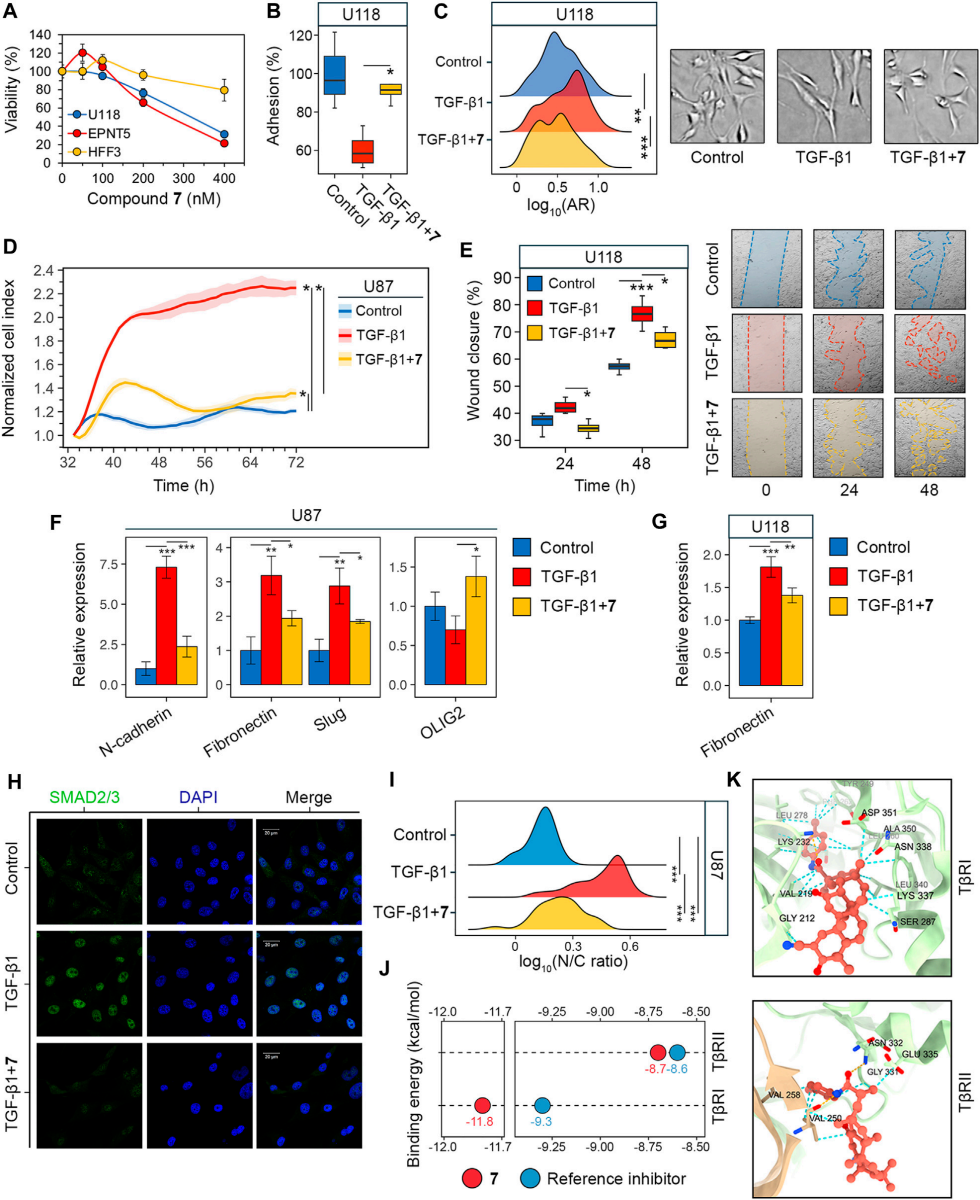
Evaluation of GMT inhibition by compound **7**. **(A)** Cytotoxicity of **7** against U118, EPNT-5 and hFF3 cells assessed by MTT assay at 48 h (n = 4). **(B–G)** The effect of **7** on the phenotypical manifestations of GMT was assessed by incubating U87 and U118 cells with TGF-β1 (50 ng/mL) and **7** (50 nM) for 48 h. **(B)** Adhesion of U118 cells to culture plastic measured by MTT assay (n = 4). **(C)** Shape distribution of U118 cells (n = 3). **(D)** Transwell migration of U87 cells (n = 4). **(E)** Wound closure on a monolayer of U118 cells (n = 3). **(F, G)** Expression of GMT markers in U87 **(F)** and U118 **(G)** cells assessed by RT-qPCR (n = 3). **(H)** Immunofluorescence staining of SMAD2/3 in U87 cells after pre-incubation with **7** for 48 h and activation with TGF-β1 for 1 h **(I)** SMAD2/3 distribution in U87 cells expressed as the ratio of nuclear (N) to cytoplasmic **(C)** intensity (n = 3, 100 cells/sample). **(J)** Binding energies of **7** to the kinase domains of TGF-β receptors type I and II (TβRI and TβRII) estimated using AutoDock Vina. For comparison, the affinities of known receptor inhibitors are given (PubChem CIDs 121411739 and 118988613 for TβRI and TβRII, respectively). **(K)** Docking structure of **7** with TβRI and TβRII visualized in ChimeraX. The orange and cyan dotted lines indicate hydrogen bonds and van der Waals interactions, respectively. Data in bar graphs are represented as mean ± standard deviation (SD). Statistical significance was calculated by comparison with the control or TGF-β1-treated group. *, **, *** indicate that *p*-values were less than 0.05, 0.01, and 0.001, respectively.

The next phase of the study aimed to investigate the impact of compound **7** on glioblastoma aggressiveness, specifically mesenchymal and stem cell traits. To ensure the reliability of the results obtained in U87 cells (Figures 2B, C), experiments were also performed in human glioblastoma U118 and mouse glioblastoma EPNT-5 cells. The MTT assay showed comparable levels of cytotoxicity of **7** in U118 and EPNT-5 cells, which were lower than that in U87 cells (IC_50_^U118^ = 309.9 nM, IC_50_^EPNT-5^ = 257.4 nM, IC_50_^U87^ = 160.6 nM) (Figures 2A, 3A). Note that the cytotoxicity of **7** against non-malignant human hFF3 fibroblasts was significantly lower than the above values: **7** at 400 nM resulted in the death of only about 21% of the cells (Figure 3A).

Similar to U87 cells, U118 cells were also sensitive to **7**: it was found that **7** at a non-toxic concentration of 50 nM effectively reversed TGF-β1-induced 40% decrease in U118 cell adhesion to culture plastic (Figure 3B). Interestingly, in contrast to U87 cells, TGF-β1 did not alter the size of U118 cells (Figure 2C), but caused them to acquire a more elongated spindle-like shape, as evidenced by a 20% increase in the ratio of the major to minor axes (aspect ratio, AR) of TGF-β1-treated cells compared to control (Figure 3C). **7** restored the shape of TGF-β1-induced cells to that of the control cells (Figure 3C).

To confirm the inhibition of GMT by **7**, we examined changes in cell motility and expression of GMT-related genes in glioblastoma cells (Zhang et al., 2017; Ouanouki et al., 2018). To assess the effect of **7** on GMT-associated motility, the transwell migration of U87 cells was monitored in a real time regimen using the xCELLigence analyzer. Figure 3D shows that the migration of U87 cells increased significantly in response to TGF-β1 up to 66 h of incubation, reaching 186% of that of control cells. The addition of **7** resulted in a drastic decrease in the motility of TGF-β1-treated U87 cells close to the level in the control (Figure 3D). Despite a difference from the control at 72 h, compound **7** suppressed transwell migration of TGF-β1-treated glioblastoma cells by 1.7-fold compared to cells stimulated with TGF-β1 alone (Figure 3D). The effect of **7** on TGF-β1-induced motility of glioblastoma cells was further independently verified using the scratch assay. As shown in Figure 3E, TGF-β1 significantly enhanced the motility of U118 cells, whereas the tested compound completely reversed or reduced the effect of TGF-β1 by 34% at 24 and 48 h, respectively. Evaluation of the levels of key GMT-associated markers in glioblastoma cells showed that TGF-β1 increased the expression of mesenchymal genes encoding N-cadherin, fibronectin, and Slug in U87 cells by 7.3-, 3.2-, and 2.9-fold, respectively, compared to untreated control, whereas the addition of **7** reduced these effects by 67.6%, 39.2%, and 35.8%, respectively, compared to TGF-β1-stimulated cells (Figure 3F). A similar inhibitory effect of **7** on fibronectin expression was observed in U118 cells (Figure 3G). In addition, qRT-PCR analysis revealed a low sensitivity of the proneural gene *OLIG2* to TGF-β1 and its 2-fold upregulation in TGF-β1-induced U87 cells treated with **7** (Figure 3F), which may also indicate the anti-GMT activity of **7**.

### 3.3 Compound 7 inhibited TGF-β1/SMAD2/3 signaling axis probably due to direct interactions with TβRI/II

In the next step of the study, the potential molecular mechanism behind the anti-GMT effect of **7** was investigated. The TGF-β1 signaling involves interaction of TGF-β1 with TGF-β receptor type II (TβRII) followed by the activation of TGF-β receptor type I (TβRI) and phosphorylation of SMAD2 and SMAD3. The latter proteins, upon binding to SMAD4, translocate to the nucleus and act as transcription factors to activate GMT-associated genes (Katsuno and Derynck, 2021). The effect of compound **7** on SMAD2/3 translocation was examined in U87 cells by indirect immunofluorescence microscopy. As shown in Figure 3H, SMAD2/3 were dispersed in the cytoplasm of untreated U87 cells, showing dim fluorescence. Stimulation with TGF-β1 for 1 h resulted in the nuclear translocation of SMAD2/3 (Figure 3H), as evidenced by a 3.3-fold increase in the nuclear-cytoplasmic ratio of their fluorescence intensity (Figure 3I). Pretreatment with **7** for 48 h prior to TGF-β1 induction nearly restored the dispersed pattern of SMAD2/3 localization in U87 cells (Figure 3H). Although the nuclear-cytoplasmic ratio of SMAD2/3 was reduced by 52% under hit compound treatment, it remained significantly different from the control group (Figure 3I). This finding is consistent with a statistically significant, but not complete, inhibition of downstream targets of the TGF-β1 signaling pathway, namely, N-cadherin, fibronectin, and Slug, in TGF-β1-activated U87 cells treated with **7** (Figure 3F). Compound **7** was further assumed to inhibit SMAD2/3 signaling by blocking TGF-β receptors. Molecular docking simulations revealed that **7** can bind to the kinase domains of TβRI and TβRII with equal or lower binding energies (∆G) than previously reported inhibitors of these receptors (Figure 3J). Compound **7** penetrated deeply into the hydrophobic pocket of the TβRI kinase domain, while remaining close to the entrance of the TβRII kinase domain (Figure 3K). As a result, it exhibited a higher affinity for TβRI than TβRII (∆G^TβRI^ = −11.8 kcal/mol vs. ∆G^TβRII^ = −8.7 kcal/mol), which was supported by greater stabilization with van der Waals forces (24 vs. 8 interactions in TβRI and TβRII, respectively) (Figure 3K). In addition, **7** formed one hydrogen bond with TβRI (Lys232; plays a critical role in TβRI kinase activity (Chaikuad and Bullock, 2016) and two hydrogen bonds with TβRII (Val250, Asn332; known amino acid residues positioning TβRII inhibitors (Verdura et al., 2022; Ye et al., 2023) (Figure 3K). Taken together, these data suggest that compound **7** inhibits GMT in glioblastoma cells through the TGF-β1/SMAD2/3 pathway.

### 3.4 Compound 7 reduced stemness of glioblastoma cells

**FIGURE 4:**
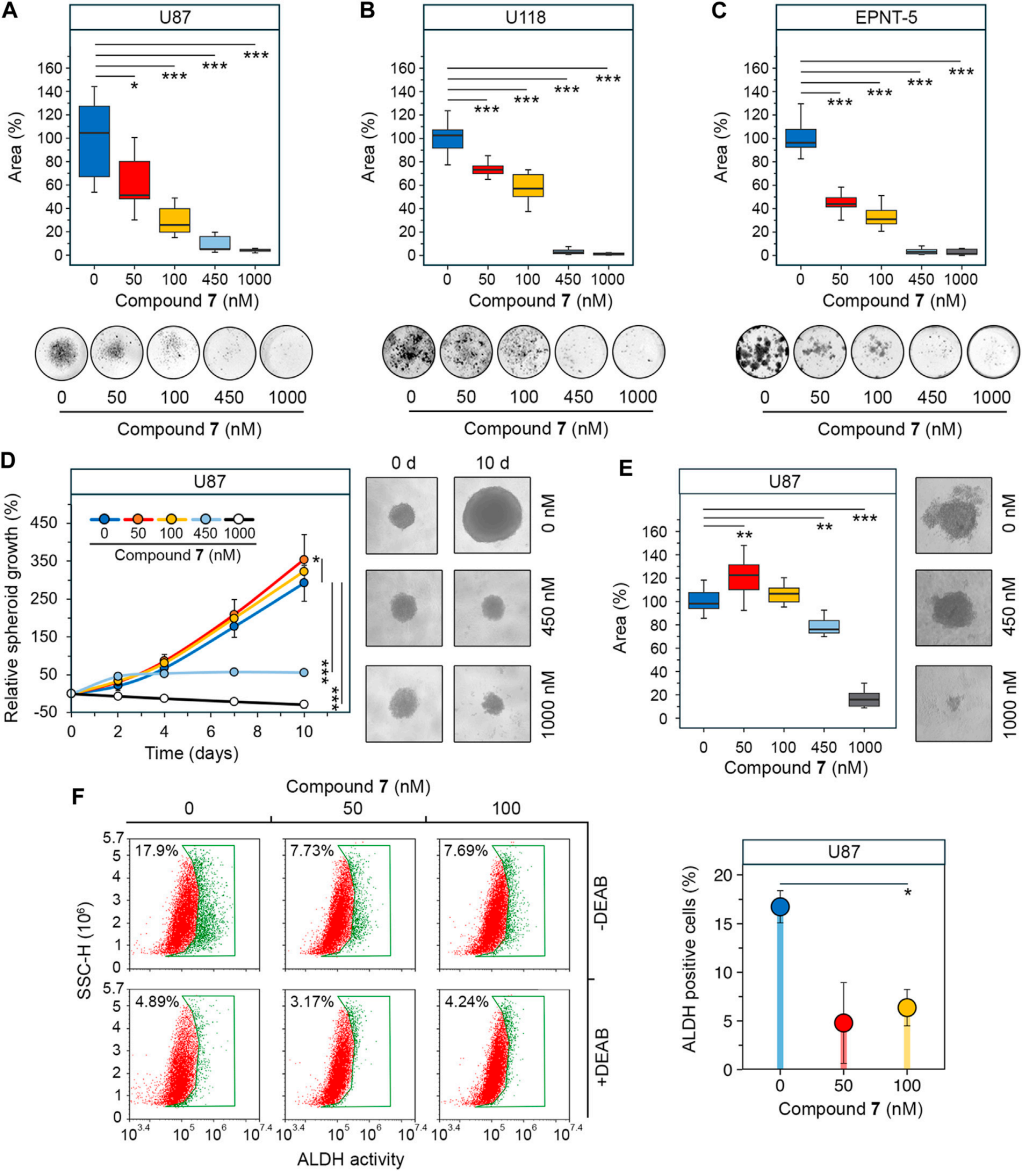
Effect of **7** on stemness of glioblastoma cells. **(A–C)** Colony forming activity of U87 **(A)**, U118 **(B)** and EPNT-5 **(C)** cells assessed after 10 days of incubation with **7** (0–1,000 nM) (n = 5). **(D)** Growth of U87 primary tumorspheres over 10 days of **7** treatment (0–1,000 nM) (n = 8). **(E)** Size of U87 secondary tumorspheres that were formed from cells of primary tumorspheres after treatment with **7** (0–1,000 nM) for 4 days (n = 8). **(F)** ALDH activity in U87 primary tumorspheres after 4 days of incubation with **7** (0–100 nM) for 4 days assessed using AldeRed staining and flow cytometry (n = 3, 10^4^ events/sample). Line graph data are represented as mean ± standard deviation (SD). Statistical significance was calculated by comparison with the untreated group. *, **, *** indicate that *p*-values were less than 0.05, 0.01, and 0.001, respectively.

GSCs are another hallmark of glioblastoma aggressiveness due to their high capacity for self-renewal and tumor initiation (Biserova et al., 2021). Colony formation assays were used as first step to assess the effect of **7** on the stemness of glioblastoma cells. The results show that increasing the concentration of **7** from 50 to 1,000 nM resulted in a gradual decrease in colony area from 51.3% to 3.9% in U87 cells after 10 days of incubation (Figure 4A). A similar trend was observed in U118 cells, although they showed a weaker response to lower concentrations of **7** (Figure 4B). The inhibition of colony formation in U118 cells was of 26.8% and 42.8% at 50 nM and 100 nM, respectively, compared to 48.7% and 74.2% inhibition observed at the same concentrations in U87 cells (Figures 4A, B). In contrast, EPNT-5 cells were more sensitive to **7** treatment, showing 56.1% and 69% inhibition at 50 nM and 100 nM, respectively, and complete abrogation of colony growth at 450 nM and 1,000 nM (Figure 4C).

Since previous studies have shown that the stemness of glioblastoma cells is increased when they are grown in tumorspheres (Lee et al., 2016), we investigated the anti-stem cell activity of **7** in a three-dimensional cell model. U87 cells were allowed to form spheres under non-adherent conditions and their growth was monitored in the presence of increasing concentrations of **7**. Figure 4D shows that untreated tumorspheres grew gradually, reaching 2.9 times their original size by day 10. The effect of **7** on tumorspheres was concentration-dependent. Surprisingly, low concentrations of **7** stimulated spheroid growth, increasing it by 21% at 50 nM (*p*-value <0.05) and 10.4% at 100 nM (not statistically significant) compared to control (Figure 4D). In contrast, high concentrations of **7** inhibited spheroid growth, stopping it after 4 days at 450 nM or completely blocking it at 1,000 nM. Notably, **7** at 1,000 nM caused tumorsphere disruption, reducing their size by 28.6% compared to control by day 10 (Figure 4D). To assess the self-renewal capacity, U87 tumorspheres were exposed to increasing concentrations of compound **7** for 4 days. Afterward, the cells were dissociated by trypsinization and left in non-adherent conditions for 3 days to form secondary tumorspheres. Similar to primary tumorspheres, **7** increased the area of secondary tumorspheres by 22.6% at 50 nM (*p*-value <0.01) and 6.6% at 100 nM (not statistically significant) (Figure 4E). In contrast, higher concentrations of **7** significantly reduced the self-renewal capacity of U87 cells. Specifically, induction with 450 nM and 1,000 nM of **7** decreased secondary tumorsphere area by 23.9% and 84.1%, respectively (Figure 4E).

Compound **7** was hypothesized to affect tumorsphere growth not only through cytotoxicity but also by interfering with stem cell behavior. Aldehyde dehydrogenases (ALDHs) induce stem cell characteristics by alleviating oxidative stress and activating the retinoic acid signaling pathway, making them a marker of GSCs (Xia et al., 2023), and ALDH activity was found to be significantly increased in tumorspheres compared to U87 cells cultured in monolayer (Supplementary Figure S2). ALDH activity was analyzed in U87 tumorspheres after 4 days of induction with low-toxic concentrations of **7** using AldeRed staining and flow cytometry. The results indicate that untreated tumorspheres had high ALDH activity, with 17.9% of cells being ALDH+ (Figure 4F). Treatment with **7** at 50 and 100 nM reduced the number of ALDH + cells by 2.3-fold compared to the control, regardless of the dose. The addition of DEAB, a selective inhibitor of ALDH, reduced the fluorescence in all samples to the basal level, demonstrating that the observed variation was due to ALDH activity (Figure 4F). Thus, the results obtained clearly indicated that the observed growth of tumorspheres at low-toxic doses of **7** was not mediated by an increase in the GSC population. The toxic profile of **7** revealed in U87 tumorspheres (Figure 4D) suggests the need for further detailed study of the bioactivity of **7** at low concentrations and confirms its eligibility for glioblastoma treatment.

### 3.5 Compound 7 increased the efficacy of temozolomide in glioblastoma cells by inducing ROS-dependent apoptosis

**FIGURE 5:**
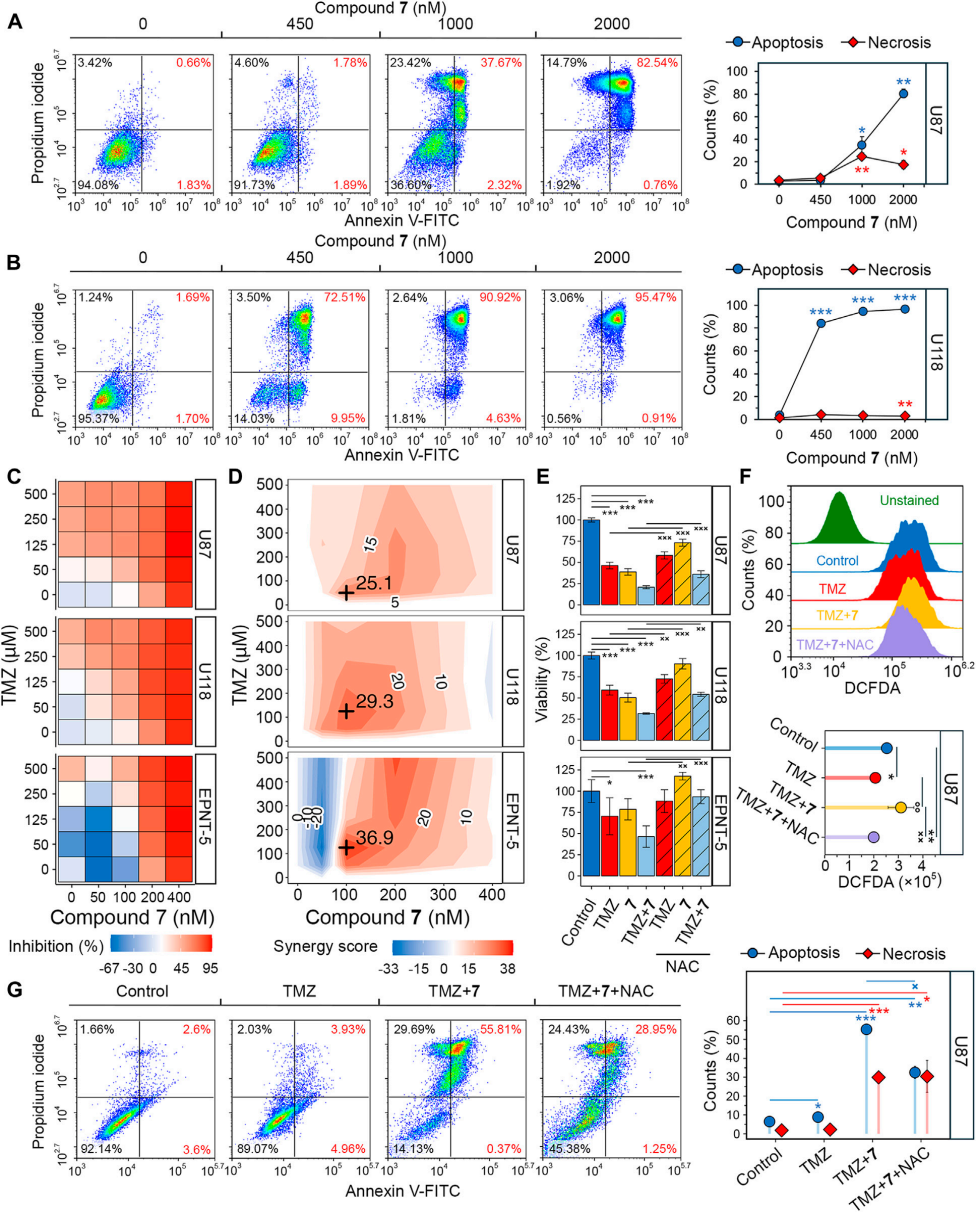
Evaluation of the synergistic effect of **7** and TMZ against glioblastoma cells. **(A, B)** Apoptosis and necrosis assessed in U87 **(A)** and U118 **(B)** cells after 48 h of incubation with **7** (0–2000 nM) using Annexin V-FITC and propidium iodide double staining and flow cytometry (n = 3, 10^4^ events/sample). **(C)** Cytotoxicity of combinations of **7** (0–400 nM) and TMZ (0–500 μM) against U87, U118 and EPNT-5 cells evaluated by MTT assay (n = 4). **(D)** Synergy score (δ) across different concentrations of **7** and TMZ in U87, U118 and EPNT-5 cells (n = 4). δ was calculated using the HSA model in the SynergyFinder + platform. Black crosses indicate maximum δ values. **(E)** The impact of ROS scavenging by NAC (2 mM) on the combined cytotoxicity of TMZ (125 μM) and **7** (100 nM) in U87, U118, and EPNT-5 cells (n = 4). Incubation time: 72 h **(F)** ROS production in U87 cells after 72 h of incubation with TMZ (125 μM) and **7** (100 nM) assessed by DCFDA staining and flow cytometry (n = 3, 10^4^ events/sample). **(G)** Apoptosis and necrosis assessed in U87 cells after 72 h of incubation with TMZ (500 μM), **7** (600 nM), and NAC (2 mM) using Annexin V-FITC and propidium iodide double staining and flow cytometry (n = 3, 10^4^ events/sample). Data in line graphs, bar graphs, and lollipop chart are represented as mean ± standard deviation (SD). Statistical significance was calculated by comparison with control (indicated by *) or the corresponding NAC-treated group (indicated by ×). */×, **/××, ***/××× indicate that *p*-values were less than 0.05, 0.01, and 0.001, respectively.

Since soloxolone tryptamide (compound **9)** has been shown to induce apoptosis in glioblastoma cells (Markov et al., 2022b), we investigated whether this form of cell death contributed to the cytotoxic effects of soloxolone *para*-methylanilide **7** in U87 and U118 cells. Annexin V-FITC and propidium iodide double staining results show that **7** induced apoptotic death in both cell lines, with U118 cells being more sensitive. The percentage of U118 cells in the right quadrants was 82.5%, 95.6%, and 96.4% at concentrations of 450 nM, 1,000 nM, and 2000 nM, respectively, compared to 3.7%, 40%, and 83.3% for U87 cells at the same concentrations (Figures 5A, B). The majority of cells undergoing apoptosis were at a late stage (upper right quadrant), likely due to the late time point of 48 h (which corresponds to our MTT evaluations). Notably, 1,000 and 2000 nM of **7** induced necrosis in 23.4% and 14.8% of U87 cells, respectively (Figure 5A). In contrast, the percentage of necrotic U118 cells did not exceed 3.5% at any concentration (Figure 5B). Thus, **7** induced the death of glioblastoma cells primarily through apoptosis.

Poor tumor cell response to TMZ is a major concern in the treatment of glioblastoma, and combination regimens with other chemotherapeutics have been developed to improve survival outcomes (Tomar et al., 2021). To evaluate whether compound **7** could enhance the efficacy of TMZ, we assessed the viability of glioblastoma cells after incubation with various dose combinations of TMZ and **7** for 72 h. TMZ exhibited low cytotoxicity at micromolar concentrations (IC_50_^U87^ = 212.7 μM, IC_50_^U118, EPNT-5^ > 500 μM), while 400 nM **7** caused almost complete death of glioblastoma cells (IC_50_^U87^ = 197.2 nM, IC_50_^U118^ = 168.6 nM, IC_50_^EPNT-5^ = 199.3 nM) (Figure 5C). The combined application of TMZ and **7** resulted in increased cytotoxicity in U87 and U118 cells compared to either compound alone. Notably, in EPNT-5 cells, a dose-dependent peculiarity in the cytotoxicity profile of **7** was revealed: the tested compound reduced TMZ-induced toxicity when used at the lowest concentration of 50 nM and increased it at higher concentrations (Figure 5C). Synergy scores (δ) were calculated for each dose combination according to the HSA model using the SynergyFinder + platform. Evaluation of drug interaction landscapes revealed a synergistic interplay between **7** and TMZ in U87 and U118 cells (Figure 5D). U118 cells showed a larger area of high synergy compared to U87 cells, resulting in a higher δ value (mean δ^U118^ = 15 vs. mean δ^U87^ = 11.6) (Figures 4C, D). The most significant synergistic effect was observed when 100 nM of **7** was combined with 50 μM of TMZ in U87 cells (δ^U87^ = 29.3) and 100 nM of **7** with 125 μM of TMZ in U118 cells (δ^U118^ = 25.1). However, no statistically significant synergy of **7** with TMZ was found in EPNT-5 cells (mean δ^EPNT-5^ = 6) due to the antagonistic effect of **7** at 50 nM on TMZ cytotoxicity (Figure 5D).

Dico et al. showed that TMZ cytotoxicity is limited in drug-resistant glioblastoma cells because it fails to promote the production of reactive oxygen species (ROS) (Dico et al., 2019). Given that compound **9** causes ROS-dependent death of glioblastoma cells (Markov et al., 2022b), we investigated whether the synergistic effect of compound **7** with TMZ was due to ROS induction. Figure 5E shows that co-incubation of U87 and U118 cells with the ROS scavenger N-acetyl-L-cysteine (NAC) for 72 h reduced the cytotoxic effect of 125 μM TMZ by 22.3% and 32.5%, respectively. The impact of NAC on the toxicity of 100 nM **7** was even more pronounced, resulting in a 56.2% and 80.3% reduction in U87 and U118 cells, respectively. Similar trends were observed for co-treatment of EPNT-5 cells with NAC and TMZ or **7**, although these data were statistically insignificant. As expected, NAC reduced the cytotoxic effect of the combination of TMZ and **7** by 19.3%, 33.1%, and 87.6% in U87, U118, and EPNT-5 cells, respectively (Figure 5E). DCFDA staining confirmed that this effect was due to ROS depletion. Co-treatment of U87 cells with TMZ and **7** for 72 h resulted in a 1.35-fold increase in ROS in U87 cells, which was significantly suppressed by NAC (Figure 5F). Interestingly, TMZ itself did not increase ROS accumulation in U87 cells, which can be explained by the high basal ROS level in U87 cells that is characteristic of TMZ-insensitive glioblastoma cells (Figures 5C, F) (Dico et al., 2019).

The mechanism of cell death induced by the combination of TMZ and **7** was further investigated using Annexin V-FITC/propidium iodide double staining. After incubation of U87 cells with 500 μM TMZ for 72 h, the apoptosis rate was comparable to that of the control group, with 9% of glioblastoma cells in the right quadrants (Figure 5G). The combination treatment of TMZ (500 μM) and **7** (600 nM) produced 6.2-fold higher activity compared to TMZ alone, resulting in 56.2% of U87 cells undergoing apoptosis. The addition of NAC reduced the observed effect by 41%, which is in line with the MTT results (Figure 5E). Notably, NAC exhibited no effect on the 30% population of necrotic cells observed with combination treatment (Figure 5G). These results demonstrate that **7** can enhance the response of glioblastoma to TMZ through a mechanism involving ROS-dependent apoptosis.

### 3.6 Compound 7 effectively suppressed U87 glioblastoma growth in a mouse xenograft model, both as a single agent and in combination with TMZ

**FIGURE 6:**
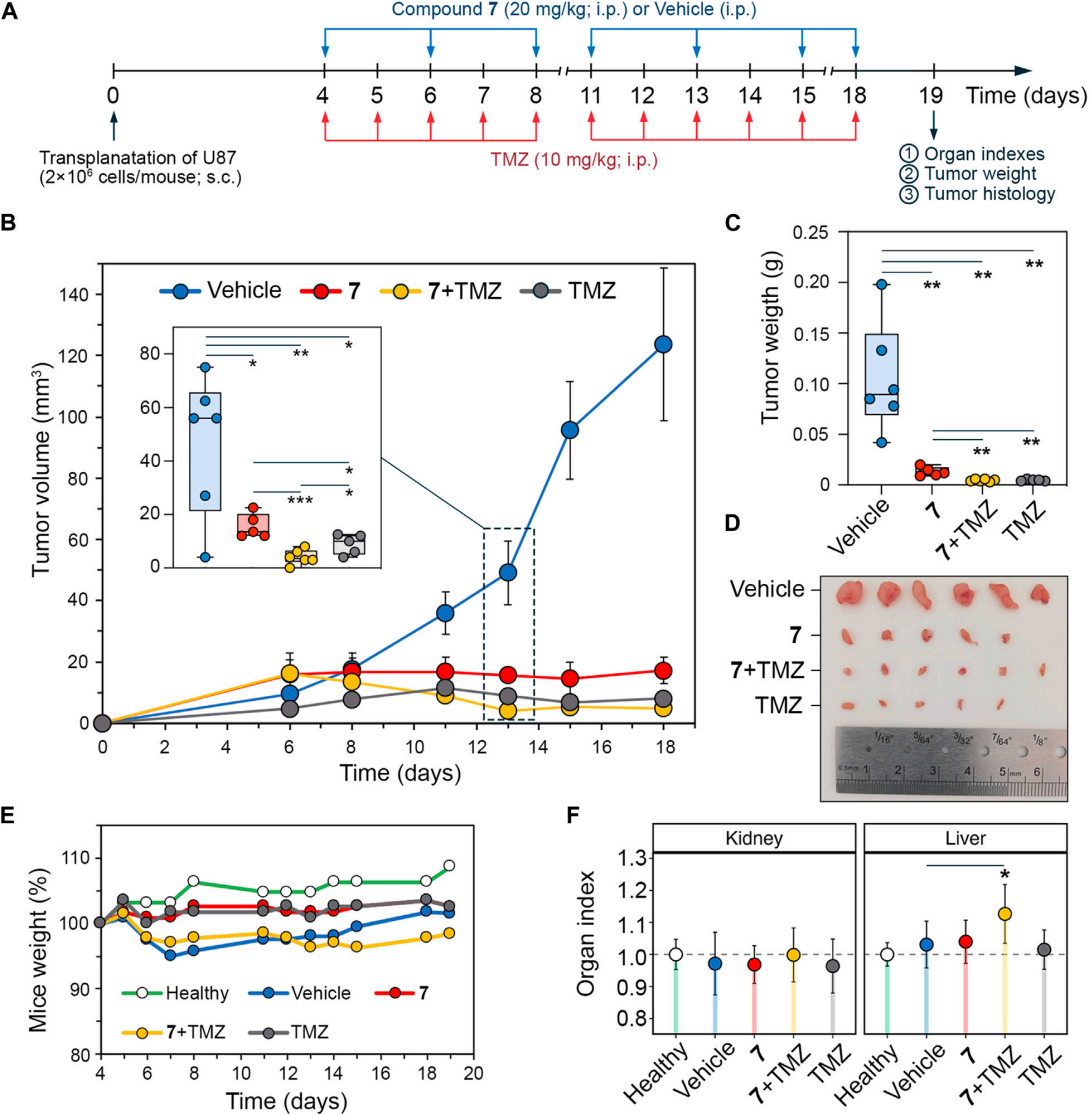
Antitumor effect of compound **7** and its combination with TMZ on U87 glioblastoma xenograft model. **(A)** Experimental setup. U87 glioblastoma cells were subcutaneously (s.c.) implanted into athymic nude mice. On day 4 after tumor transplantation mice were treated with compound **7** (20 mg/kg, i.p., n = 5), TMZ (10 mg/kg, i.p., n = 5) or their combination at the same doses and administration route (n = 6). Compound **7** was administered three times per week for a total of seven injections. TMZ was administered daily, except weekends, for a total of eleven injections. Vehicle-treated mice were used as control (n = 6). Mice were sacrificed on day 19 after tumor transplantation, and material (tumor nodes, livers, and kidneys) was collected for further analysis. **(B–D)** Compound 7 effectively suppresses the growth of U87 glioblastoma. Tumor volumes **(B)**, tumor weights **(C)**, and primary tumor nodes **(D)** of U87 glioblastoma without treatment and after administration of **7**, TMZ or their combination. The insert shows tumor volumes on day 13 of tumor growth. **(E, D)** Mice weights **(E)** and relative organ indices **(F)** of U87 glioblastoma-bearing mice without treatment and after administration of **7**, TMZ or their combination.

To verify the pronounced anti-glioblastoma potency of **7** and its potentiating effect on TMZ observed *in vitro*, athymic nude mice bearing subcutaneously implanted U87 glioblastoma were treated intraperitoneally with **7** (20 mg/kg), TMZ (10 mg/kg) or their combination at the same doses (Figure 6A). Compound **7** was administered three times per week for a total of 7 injections, whereas TMZ was injected daily except weekends for a total of 11 times. Starting on day 11, **7** was found to obviously suppress tumor growth, resulting in 2.1-, 3.2-, 6.6-, and 7.2-fold reduction in tumor volume compared to the vehicle group on days 11, 13, 15, and 18, respectively (Figure 6B), demonstrating comparable efficacy to TMZ. Notably, on day 13, the combination scheme exceeded the effects of **7** and TMZ administered alone by 3.9- and 2.2-fold, respectively (Figure 6B), which is consistent with the ability of **7** to enhance cytotoxicity of TMZ observed *in vitro* (Figures 5C, D). Nevertheless, with subsequent injections, the antitumor efficacy of the combination of **7** with TMZ becomes comparable to that of TMZ injected alone, achieving 17.9- and 25.8-fold reductions in tumor volume compared to the vehicle group on days 15 and 18, respectively (Figure 6B). At the end of the experiment, in addition to the effect on tumor volume, **7**, TMZ and their combination resulted in an 8-, 21.9- and 22.5-fold reduction in tumor weight, respectively, compared to the vehicle (Figure 6C). Analysis of body weight and organ indices showed no toxic effects of **7** and TMZ injected alone, while their combination resulted in a slight reduction in body weight and a statistically significant increase in liver index, but since these changes did not exceed 10% compared to the vehicle group, the treatment regimen used was considered well tolerated (Figures 6E, F).

**FIGURE 7:**
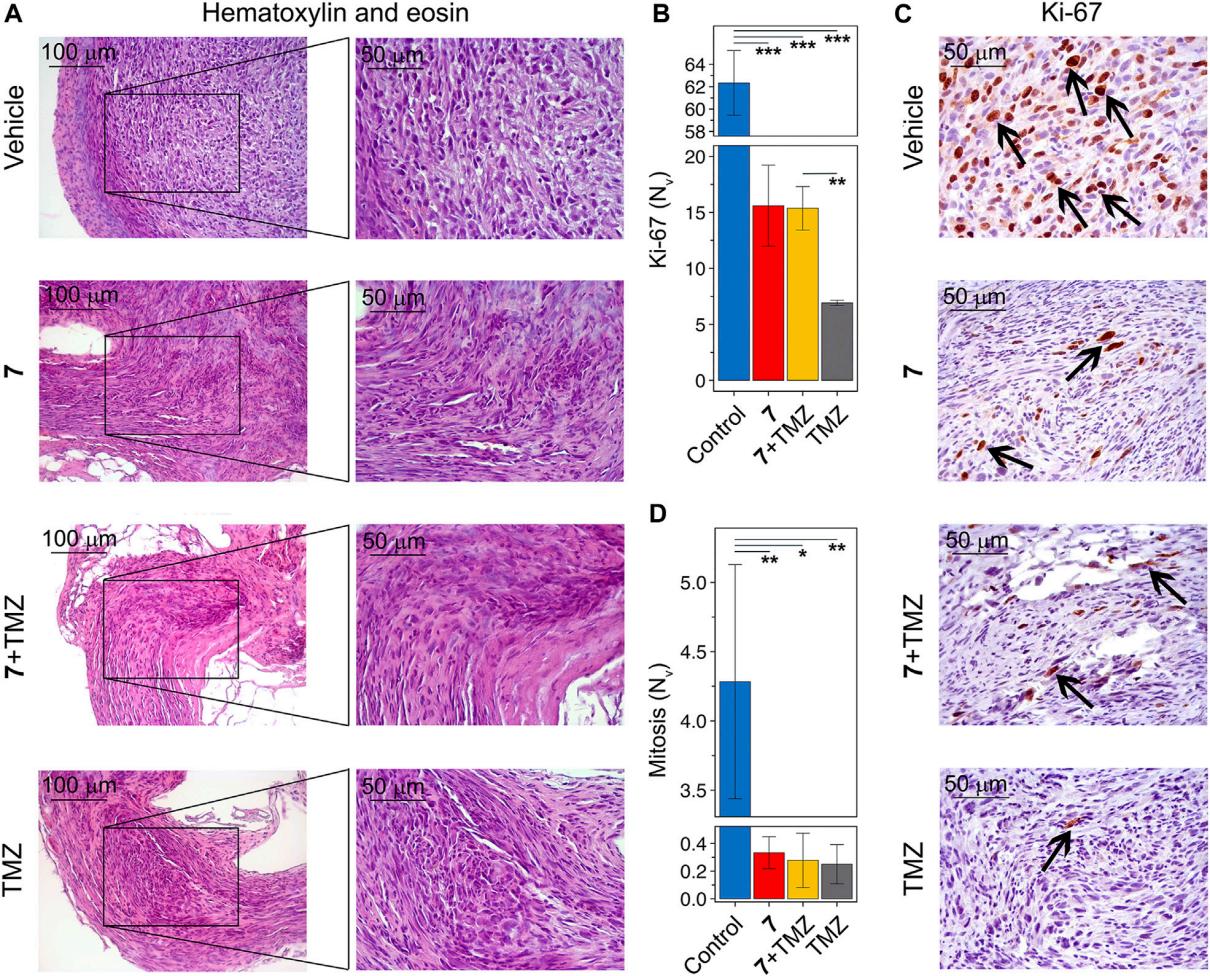
Histological structure of U87 glioblastoma without treatment and after administration of **7**, TMZ or their combination. **(A)** Representative histological images of U87 glioblastoma in control and experimental groups. The black boxes show areas that were examined further at a higher magnification. Hematoxylin and eosin staining. Original magnification ×200 (left panel) and ×400 (right panel). **(B)** The numerical density (Nv) of tumor cells in the state of mitosis (n = 5–6). **(C)** Immunohistochemical staining of U87 glioblastoma with primary anti-Ki-67 antibodies. The black arrows indicate Ki-67 positive cells. Original magnification ×400. **(D)** The numerical density (Nv) of Ki-67 positive cells (n = 3).

Histologically, tumor nodes of U87 glioblastoma in the vehicle-treated group were represented by sheets and bundles of undifferentiated polygonal to spindle-shaped cells with variable amounts of fibrovascular stroma (Figure 7A). The cells were characterized by centrally located round to irregular nuclei with punctate chromatin and 1-2 nucleoli and moderate nuclear to cytoplasmic ratio. Some cells, amounting to 4.3 ± 0.8 per field of view, contained mitotic events. Along the periphery of the tumor node, tumor cells became highly elongated forming a demarcating pseudocapsule. Immunohistochemical staining of U87 glioblastoma with primary anti-Ki-67 antibody indicates that tumor tissue of the vehicle-treated group is characterized by the pronounced proliferative activity, expressing in a large amount of Ki-67-positive cells in the tumor structure with a numerical density of 62.3 ± 2.9 in the square unit of tumor section (Figures 7B, C). A significant 13- to 17-fold reduction in the number of mitoses was observed in tumor nodes treated with any of the regimens used compared to vehicle (Figure 7D). Consistent with this, tumors following compound **7** and combination therapy were characterized by a significant 4-fold reduction in the numerical density of Ki-67-positive cells compared to the vehicle-treated group (Figure 7B). TMZ was found to result in an almost complete disappearance of Ki-67 positive cells in the tumor tissue, expressed by a 9-fold decrease in their numerical density compared to vehicle. Surprisingly, despite a similar effect on the number of mitoses in the tumor, the effect of TMZ on Ki-67 positive cells was more pronounced than that of **7** administered alone or in combination with TMZ (Figure 7B).

**FIGURE 8:**
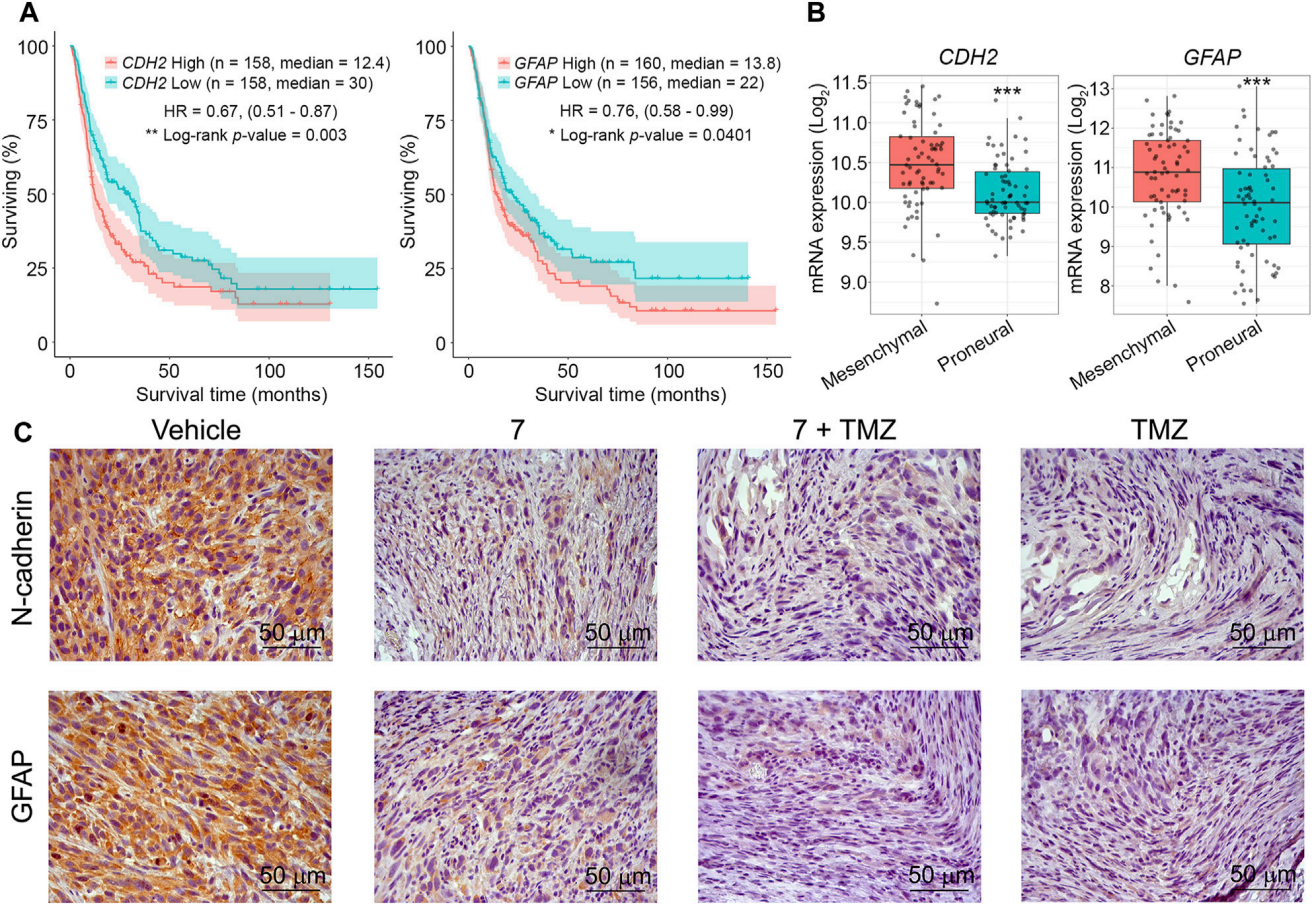
The effect of **7**, TMZ or their combination on the expression of N-cadherin and GFAP in U87 glioblastoma. **(A)** Survival of patients with recurrent glioma depending on the expression levels of *CDH2* and *GFAP* (CCGA database). **(B)** Expression levels of *CDH2* and *GFAP* in mesenchymal- and proneural-type glioblastoma (REMBRANDT database). *** indicates that *p*-values were less than 0.001. **(C)** Representative images of U87 glioblastoma immunohistochemically stained with primary anti-N-cadherin (upper panel) and anti-GFAP (lower panel) antibodies. Original magnification ×400.

Finally, the effect of **7** and its combination with TMZ on the expression of markers associated with glioblastoma aggressiveness was evaluated. We selected N-cadherin, a mesenchymal-type marker that is sensitive to **7** in U87 cells *in vitro* (Figure 3F), and glial fibrillary acidic protein (GFAP), which plays an important regulatory role in glioma cell motility (Uceda-Castro et al., 2022), as markers of interest. Additional analysis of the Chinese Glioma Genome Atlas (CCGA) showed that high expression levels of CDH2 and GFAP genes encoding these proteins were significantly associated with poor survival in patients with recurrent glioma (Figure 8A), and according to the Repository for Molecular BRAin Neoplasia DaTa (REMBRANDT), their overexpression is characteristic of mesenchymal-type glioblastoma cells compared to less aggressive proneural-type glioblastoma cells (Figure 8B). Immunohistochemical staining of vehicle-treated U87 glioblastoma sections with anti-N-cadherin and anti-GFAP antibodies showed high expression of both markers, whereas administration of **7**, its combination with TMZ, and TMZ alone significantly reduced their levels, with the most pronounced effect observed in mice treated with TMZ or combined therapy (Figure 8C). These results independently confirm the ability of **7** to inhibit N-cadherin expression in glioblastoma cells observed *in vitro* (Figure 3F) and are consistent with recently published data on the ability of TMZ to inhibit GFAP expression in U87 glioblastoma in a murine xenograft model (Bakhtiyari-Ramezani et al., 2024).

Thus, the results of the *in vivo* experiment confirmed the pronounced anti-glioblastoma potential of **7** and its ability to enhance the antitumor effect of TMZ, but this enhancement in the mouse model was short-term, which is probably related to the peculiarities of pharmacokinetics and biodistribution of **7** and requires further careful study.

## 4 Discussion

Glioblastoma is the most common primary brain tumor, with an annual incidence of 3–4 cases per 100,000 person-years (Grochans et al., 2022). The current standard of care is maximal safe resection followed by radiotherapy and TMZ chemotherapy (Rodríguez-Camacho et al., 2022). However, its efficacy is limited and recurrence is practically inevitable due to two main factors. First, glioblastoma infiltrates the brain tissue, making complete resection impossible (Seker-Polat et al., 2022). Second, therapeutic pressure promotes the survival of radio- and chemoresistant tumor cells, leading to difficult-to-treat relapses (Wu W. et al., 2021). Therefore, novel therapeutic approaches are needed to combat glioblastoma invasion and drug resistance.

Natural metabolites with multi-target effects are considered a promising source of new drug candidates for glioblastoma therapy. To date, a large number of natural compounds, including sesquiterpene lactones (Hsu et al., 2024), endocannabinoids (Duzan et al., 2023), quinones (Liu et al., 2024), flavonoids (Majchrzak-Celińska and Studzińska-Sroka, 2024), terpenoids (Wang and Hu, 2023) and others, have been reported to have pronounced anti-glioblastoma potential. Several pentacyclic triterpenoids (PTs) have shown promising anti-glioblastoma activity in preclinical studies, including ursolic acid (Wang et al., 2012; Lu et al., 2014; Bergamin et al., 2017), asiatic acid (Kavitha et al., 2015; Thakor et al., 2017), oleanolic acid (Fujiwara et al., 2011), and celastrol (Boridy et al., 2014; Cha et al., 2019), which raises the prospect of further PTs derivatization for the development of effective anti-glioblastoma drugs (Bache et al., 2014; Ciftci et al., 2021; Tsai et al., 2021; Markov et al., 2022b; Markov et al., 2023; Tsai et al., 2023). The addition of a cyanoenone group to the A-ring of oleanolic and 18βH-glycyrrhetinic acids led to the development of bardoxolone (CDDO) and soloxolone methyl (SM), semisynthetic triterpenoids with pronounced antitumor potential (Logashenko et al., 2011; Borella et al., 2019). To enhance their applicability for brain disorder therapy, amide derivatives of CDDO and SM with improved blood-brain barrier permeability and high anti-glioblastoma potency were synthesized (Stack et al., 2010; Markov et al., 2022b). The trifluoroethylamide derivative of CDDO (CDDO-TFEA) was shown to reduce the viability of GBM8401 and U87 cells via AKT-mediated transcriptomic regulation of key genes associated with cell cycle, apoptosis, and autophagy (Tsai et al., 2021; Tsai et al., 2023). The soloxolone tryptamide (compound **9**) inhibited the growth of U87 and U118 cells both *in vitro* and *in vivo* by inducing intrinsic apoptosis and inhibiting angiogenesis (Markov et al., 2022b; Markov et al., 2023). Therefore, PTs represent a promising platform for the development of novel anti-glioblastoma drugs.

The invasion of glioblastoma cells into brain tissue is facilitated by glial-mesenchymal transition (GMT). This process represents a transformation of glioblastoma cells from a proneural to a mesenchymal subtype that includes morphological changes, loss of cell-cell contacts and enhancement of cell-ECM adhesion, increased motility and transcriptomic alterations (Joseph et al., 2014; Ouanouki et al., 2018; Xu et al., 2022). Although targeting GMT-associated pathways has shown promise, it has not yielded significant results in the clinical setting, indicating the need for further optimization of this strategy (Lai et al., 2024). Although PTs have been shown to inhibit glioblastoma motility (Guimarães et al., 2017; Thakor et al., 2017; Li et al., 2018; Sun et al., 2021), their effects on the molecular characteristics of GMT have not been characterized. To our knowledge, only two PT saponins, raddeanin A and tubeimoside-1, have been reported to downregulate the basal level of GMT markers along with their suppressive effect on glioblastoma cell migration and invasion in the absence of GMT inducers (Cao et al., 2019; Wu B. et al., 2021). Given that mesenchymal-like features in glioblastoma cells can be significantly enhanced by various tumor-related processes, such as hypoxia, necrosis and inflammation (Kim et al., 2021), we decided to investigate the effect of soloxolone amides on GMT induced by transforming growth factor beta (TGF-β), a known GMT activator produced by microglia, tumor-associated macrophages, and tumor cells themselves in the glioblastoma microenvironment (Golán-Cancela and Caja, 2024). Consistent with previous data, soloxolone amides inhibited GMT-associated processes in U87 cells, including changes in cellular morphology and adhesiveness (Figures 2B–D). Furthermore, hit compound **7** bearing the *p*-tolylamide group, reduced motility and the expression of the mesenchymal markers N-cadherin, fibronectin, and Slug in U87 and U118 cells induced with TGF-β1 (Figures 3D–G). The demonstrated ability of **7** to upregulate *OLIG2* in TGF-β1-stimulated U87 cells (Figure 3F) may, on the one hand, also attest to its anti-GMT potency, as OLIG2 plays an important pro-neural function in the development of the central neural system and is a marker for the proneural glioblastoma subtype (Popova et al., 2014), but on the other hand, the observed, albeit weak, increase in *OLIG2* expression under compound **7** treatment may also demonstrate the induction of a compensatory response of glioblastoma cells to the investigated triterpenoid. According to Szu et al., upregulation of OLIG2 is characteristic of glioma cells and is associated with their high proliferative potential, and pharmacological inhibition of OLIG2 significantly improved the survival of mice with high-grade glioma and medulloblastoma (Szu et al., 2023). Considering that the upregulation of *OLIG2* by compound **7** was still weakly expressed (Figure 3F), this effect of the investigated triterpenoid was not accompanied with enhanced cell proliferation.

Upon binding to TGF-β, the type II receptor (TβRII) on the surface of glioblastoma cells recruits and phosphorylates the type I receptor (TβRI). This activation leads to phosphorylation of SMAD2 and SMAD3, which then complex with SMAD4 and translocate to the nucleus to induce transcription of genes regulating GMT (Golán-Cancela and Caja, 2024). Compound **7** was shown to block SMAD2/3 nuclear translocation in U87 cells upon TGF-β induction (Figures 3H, I). This activity is similar to that previously reported by Wang et al. for ursolic acid, which effectively inhibited SMAD2/3 phosphorylation in U251 cells (Wang et al., 2012). Molecular docking simulations revealed that compound **7** can form stable complexes with TβRI and TβRII (Figures 3J, K), and these interactions may underlie the observed modulatory effect of **7** on the nuclear-to-cytoplasmic ratio of SMAD2/3. Notably, the limonoid isotoosendanin, which is structurally similar to PTs, binds to the kinase domain of TβRI in a manner similar to **7** by interacting with Lys 232, thereby reducing the association of TβRI with SMAD2/3 (Zhang et al., 2023). In addition, oleanolic, ursolic, and asiatic acids, as well as the triterpene saponin 20(S)-ginsenoside Rg3, have been shown to bind directly to TβRI, but the precise determinants governing these interactions remain to be elucidated (Yoshimura et al., 2003; Zhang et al., 2022; Xu et al., 2023). While a computer simulation suggests that **7** may interact with TβRI, further experimental verification is needed to confirm this activity.

Glioblastoma stem cells (GSCs) play a critical role in therapeutic resistance due to their ability to withstand the effects of radiation and chemotherapy in a quiescent state and subsequently resume tumor growth (Xie et al., 2022). Various GSC-targeting therapeutics are under development, and some have entered clinical trials (Sahoo et al., 2024). In the field of PT-based drug candidates, β-escin and 7β-22 dihydroxyhopane have been shown to selectively target proliferation and induce apoptosis in GSCs but not in differentiated glioblastoma cells. In addition, they block GSC stemness, as evidenced by the inhibition of tumorsphere formation, self-renewal capacity, and expression of stem cell markers (Harford-Wright et al., 2016; Kim et al., 2022). Consistent with these data, compound **7** exhibited anti-stem cell activity by inhibiting colony formation (Figures 4A–C), tumorsphere growth and renewal (Figures 4D, E), and the activity of the stem cell marker aldehyde dehydrogenase (ALDH) (Figure 4F). However, in contrast to β-escin and 7β-22 dihydroxyhopane, **7** not only affected GSCs but also showed cytotoxicity against differentiated glioblastoma cells (Figures 2A, 3A). The effect of **7** on the growth of U87 tumorspheres, both primary and secondary, was, surprisingly, dose-dependent. At a low toxic concentration of 50 nM, **7** increased the tumorsphere size compared to the control, whereas its higher, more toxic concentrations of 450 and 1,000 nM resulted in a significant reduction of spheroid growth up to its complete blockage (Figures 4D, E). Given the observed decrease in stem cell-related ALDH activity in U87 cells treated with **7** at 50 nM (Figure 4F), we speculate that **7** at low concentrations may induce the transition of GSCs into differentiated tumor cells with greater proliferative activity by suppressing their stemness, but this effect may be counteracted by the cytotoxicity of **7** at higher concentrations (Lan et al., 2017). The observed peculiarities in the effect of **7** on three-dimensional tumor growth suggest the need for further detailed studies on its pharmacokinetics.

Temozolomide (TMZ) is a first-line drug for glioblastoma that interferes with tumor cell proliferation by alkylating DNA guanines (Teraiya et al., 2023). Intrinsic and acquired resistance to TMZ remains a major challenge in the treatment of glioblastoma. One approach to address this issue is to combine TMZ with cytotoxic compounds that have alternative mechanisms for inducing cell death (Tomar et al., 2021). The results show that compound **7** has a higher cytotoxic potential and pro-apoptotic activity against glioblastoma cells *in vitro* than TMZ (Figures 5A, E, G), whereas in the murine U87 xenograft model, these compounds exhibited comparable levels of tumor growth inhibition (Figures 6B–D), accompanied by a marked reduction in the number of mitosis and Ki-67 positive cells (Figure 7B). The combined application of **7** and TMZ resulted in a synergistic reduction of viability and induction of apoptosis in glioblastoma cells *in vitro* (Figures 5C, D), which was partially verified in the xenograft model. In the mid-term (13 days after tumor transplantation), co-administration of **7** and TMZ resulted in a greater suppression of tumor growth compared to either agent administered alone (Figure 6B). However, at the end of the experiment, the tumor inhibitory effect of the combination therapy was comparable to that of TMZ, suggesting the need for additional pharmacokinetic and distribution studies to determine the appropriate pharmacological form, route, and schedule of administration of **7** to prolong its synergistic effect with TMZ *in vivo*.

Reactive oxygen species (ROS) play a dual role in glioblastoma. While ROS promote tumor cell proliferation, their excessive accumulation can damage cell lipids, proteins, and DNA, leading to apoptosis (Campos-Sandoval et al., 2021; Chien et al., 2021). Because of this double-edged effect, both antioxidant (McConnell et al., 2018; Tai et al., 2021) and prooxidant (Yuan et al., 2012; Grogan et al., 2014; Yin et al., 2014) compounds have been shown to sensitize glioblastoma cells to TMZ. With respect to PTs, Barbarisi et al. found that boswellic acid had a synergistic effect with TMZ while reducing ROS, lipid peroxides, and nitric oxide in glioblastoma cells (Barbarisi et al., 2019). In contrast to this study, the cytotoxicity of **7**, used both alone and in combination with TMZ, was highly dependent on the induction of ROS (Figures 5E, F), as ROS depletion obviously reduced this activity (Figure 5E), and, moreover, decreased the pro-apoptotic effect of the combination regimen (Figure 5G). These results are consistent with our previous in-depth investigation of the mechanisms of cell death induced by soloxolone amides (Markov et al., 2022b). It is noteworthy that some glioblastoma cells undergo necrosis after incubation with **7** (Figures 5A, G). Lu et al. previously demonstrated that ursolic acid induces necrosis in TMZ-resistant glioblastoma cells by opening the mitochondrial permeability transition pore (MPTP) in a ROS-dependent manner, resulting in decreased ATP levels and prevention of the apoptotic process (Lu et al., 2014). Given that necrosis is involved in glioblastoma progression (Markwell et al., 2022), further research is needed to investigate the therapeutic role and mechanism of compound **7**-induced necrosis.

Comparing the results described above with the results of our recent studies on the anti-glioblastoma potential of compound **9** (also known as soloxolone tryptamide) (Markov et al., 2022b; Markov et al., 2023), it can be concluded that compound **7**, despite a comparable effect on glioblastoma cell morphology and adhesion as compound **9** (Figures 2B, C), exhibits more pronounced antitumor properties because:

• **7** is more cytotoxic (Figure 2A);
• **7** shows a stronger suppressive effect on spheroid growth (**7** caused a large-scale reduction of spheroids at 1 µM (Figure 4D), whereas **9** showed a similar effect only at 4 µM (Markov et al., 2023));
• **7** has a more pronounced anti-glioblastoma effect *in vivo* (at the end of the experiment, **7** reduced tumor weight 8-fold compared to the vehicle-treated group (Figure 6C), whereas **9**, administered at a similar dose and schedule, reduced this parameter only 3.4-fold (Markov et al., 2022a)).

In addition, our findings significantly expand our knowledge of the anti-glioblastoma potential of cyanoenone-containing triterpenoids, as this study is the first to demonstrate their ability to inhibit the TGF-β-induced aggressive phenotype of glioblastoma cells.

Despite the evidence for potent anti-glioblastoma properties of compound **7** *in vitro* and *in vivo*, the present study has several limitations. First, the direct interaction of **7** with TβRI/II requires further experimental verification by surface plasmon resonance or affinity labeling competition assays. Second, for a more complete understanding of the inhibitory effect of **7** on the TGF-β/Smad signaling axis, it is necessary to additionally evaluate its effect on nuclear translocation not only of SMAD2/3 but also of SMAD4, because according to Fink et al. SMAD4, although not necessarily required for SMAD2/3 translocation to the nucleus, is critical for the TGF-β-associated transcriptional response (Fink et al., 2003). Third, given the importance of oxidative stress in compound **7**-induced glioblastoma cell death, the effect of **7** on ROS generation at early time points should be evaluated. Fourth, as mentioned above, a detailed study of the pharmacokinetics and biodistribution of **7** is needed to more accurately assess the efficacy of combined therapy of glioblastoma with **7** and TMZ *in vivo*.

## 5 Conclusion

Our study demonstrated that soloxolone *para*-methylanilide **7** effectively inhibited the viability, invasiveness, and stemness of glioblastoma cells. Importantly, **7** reversed GMT through the TGF-β1/SMAD2/3 pathway and enhanced the cytotoxicity of TMZ by inducing ROS-dependent apoptosis. These effects make **7** a potential candidate for inclusion in combination treatment regimens for glioblastoma.

## Data Availability

The original contributions presented in the study are included in the article/Supplementary Material, further inquiries can be directed to the corresponding author.
