# Supplementary material for: Soloxolone para-methylanilide effectively suppresses aggressive phenotype of glioblastoma cells including TGF-β1-induced glial-mesenchymal transition in vitro and inhibits growth of U87 glioblastoma xenografts in mice
Source: Front Pharmacol. 2024 Jul 29;15:1428924. doi: 10.3389/fphar.2024.1428924 (PMC11317440; doi:10.3389/fphar.2024.1428924)
Supplement: Supplementary file 1 [file DataSheet1.pdf]

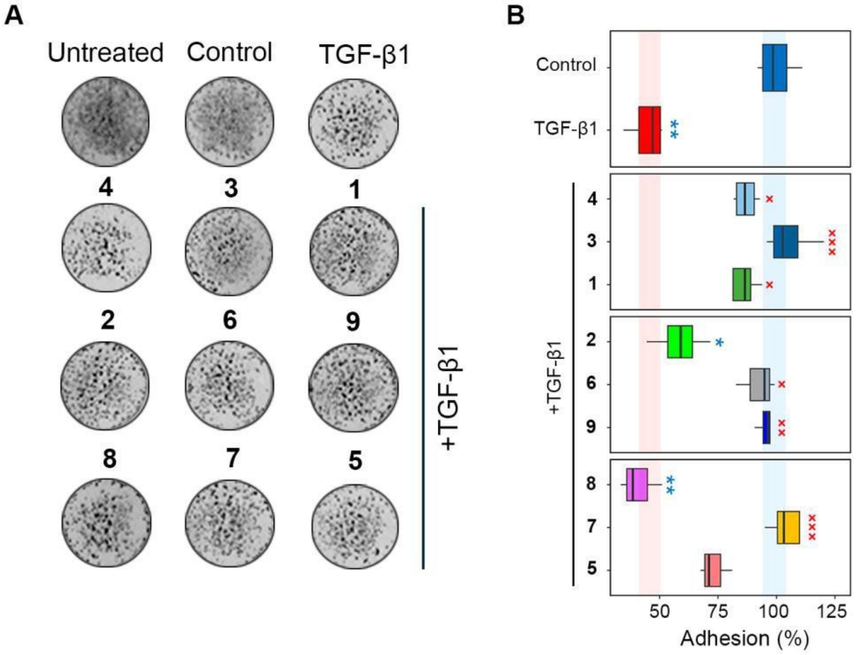

Figure S1. Evaluation of the effects of soloxolone amides on adhesion of glioblastoma cells to culture plate. (**A**, **B**) U87 cells were induced with TGF-β1 (50 ng/mL) and soloxolone amides (non-toxic concentrations) for 48 h. Cells were then treated with 5% TrypLE Express for 3 min and then washed three rimes with PBS. (**A**) Photographs of adherent U87 cells stained with crystal violet. (**B**) Number of adherent U87 cells assessed with MTT assay. Statistical significance was calculated by comparison with the control (marked by *) or TGF-β1-treated group (marked by ×). */×, **/××, ***/××× indicate that *p*-values were less than 0.05, 0.01, and 0.001, respectively.

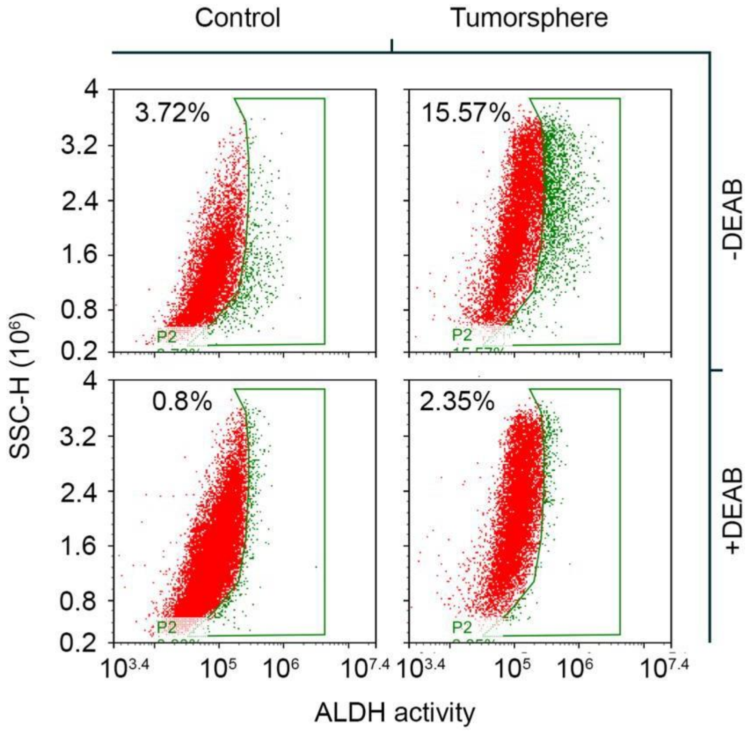

Figure S2. ALDH activity in U87 cells cultured as monolayers and primary tumorspheres assessed by AldeRed staining and flow cytometry (n = 3, 10^4 events/sample).
